# Cross-validation of SARS-CoV-2 responses in kidney organoids and clinical populations

**DOI:** 10.1172/jci.insight.154882

**Published:** 2021-12-22

**Authors:** Louisa Helms, Silvia Marchiano, Ian B. Stanaway, Tien-Ying Hsiang, Benjamin A. Juliar, Shally Saini, Yan Ting Zhao, Akshita Khanna, Rajasree Menon, Fadhl Alakwaa, Carmen Mikacenic, Eric D. Morrell, Mark M. Wurfel, Matthias Kretzler, Jennifer L. Harder, Charles E. Murry, Jonathan Himmelfarb, Hannele Ruohola-Baker, Pavan K. Bhatraju, Michael Gale, Benjamin S. Freedman

**Affiliations:** 1 Department of Medicine;; 2 Division of Nephrology;; 3 Kidney Research Institute;; 4 Institute for Stem Cell and Regenerative Medicine;; 5 Department of Laboratory Medicine and Pathology;; 6 Division of Cardiology;; 7 Center for Cardiovascular Biology;; 8 Center for Innate Immunity and Immune Disease, Department of Immunology;; 9 Department of Biochemistry; and; 10 Department of Oral Health Sciences, School of Dentistry, University of Washington School of Medicine, Seattle, Washington, USA.; 11 Division of Nephrology, Department of Internal Medicine, University of Michigan, Ann Arbor, Michigan, USA.; 12 Translational Research, Benaroya Research Institute, Seattle, Washington, USA.; 13 Division of Pulmonary, Critical Care and Sleep Medicine, University of Washington School of Medicine, Seattle, Washington, USA.; 14 Department of Computational Medicine and Bioinformatics, University of Michigan, Ann Arbor, Michigan, USA.; 15 Sana Biotechnology, Seattle, Washington, USA.; 16 Department of Bioengineering, University of Washington, Seattle, Washington, USA.

**Keywords:** COVID-19, Nephrology, Genetic diseases, Molecular pathology, iPS cells

## Abstract

Kidneys are critical target organs of COVID-19, but susceptibility and responses to infection remain poorly understood. Here, we combine SARS-CoV-2 variants with genome-edited kidney organoids and clinical data to investigate tropism, mechanism, and therapeutics. SARS-CoV-2 specifically infects organoid proximal tubules among diverse cell types. Infections produce replicating virus, apoptosis, and disrupted cell morphology, features of which are revealed in the context of polycystic kidney disease. Cross-validation of gene expression patterns in organoids reflects proteomic signatures of COVID-19 in the urine of critically ill patients indicating interferon pathway upregulation. SARS-CoV-2 viral variants alpha, beta, gamma, kappa, and delta exhibit comparable levels of infection in organoids. Infection is ameliorated in *ACE2–/–* organoids and blocked via treatment with de novo–designed spike binder peptides. Collectively, these studies clarify the impact of kidney infection in COVID-19 as reflected in organoids and clinical populations, enabling assessment of viral fitness and emerging therapies.

## Graphical abstract

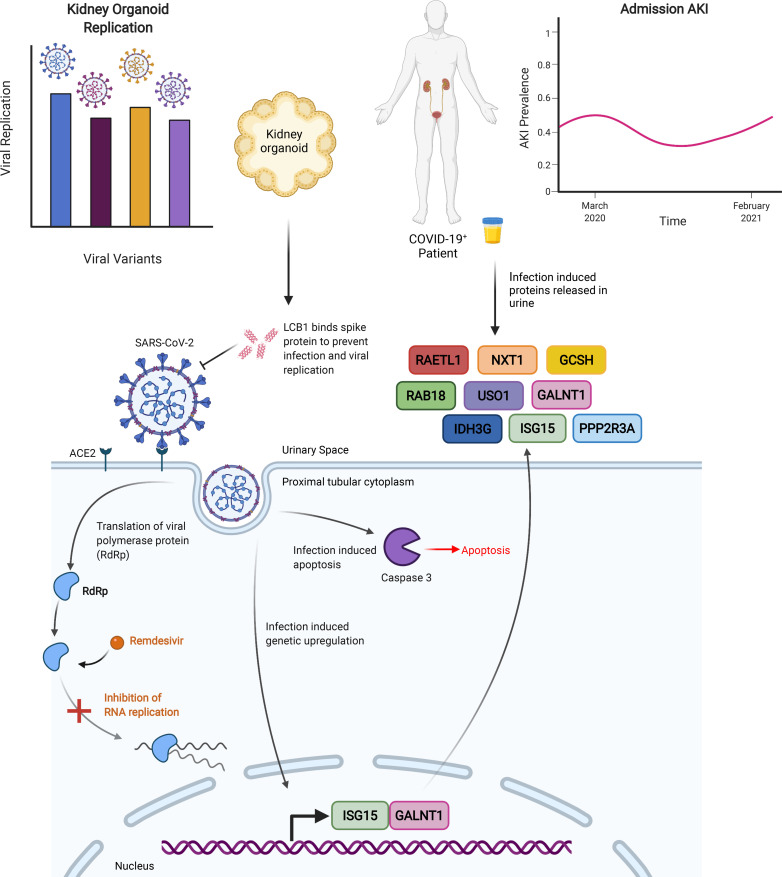

## Introduction

Severe acute respiratory syndrome coronavirus 2 (SARS-CoV-2), first detected at the end of 2019 in the Hubei province of China, has spread worldwide, causing the coronavirus disease COVID-19 (1). Despite substantial progress and therapeutic innovation, COVID-19 continues to spread and impact the globe. SARS-CoV-2 belongs to the viral family Coronaviridae, in which 3 new coronaviruses have emerged from animal reservoirs in the past 2 decades causing serious illness and death: SARS-CoV, Middle East respiratory syndrome coronavirus (MERS-CoV), and now SARS-CoV-2 (2). In addition to respiratory distress, patients with COVID-19 exhibit systemic symptoms that involve the kidneys, similarly to previous SARS-CoV and MERS-CoV outbreaks (3–5). Chronic kidney disease and its causes such as hypertension and diabetes are noted risk factors for developing severe COVID-19 disease, and COVID-19 patients frequently develop acute kidney injury (AKI) (3, 6). Angiotensin-converting enzyme 2 (ACE2) is the primary entry receptor for SARS-CoV-2 in many experimental models and is strongly expressed in the proximal tubular epithelial cells of the kidney, implicating the kidney as a target for SARS-CoV-2 infection (7–11). Multiple autopsy reports have suggested SARS-CoV-2 infection of the kidney, and several groups have isolated SARS-CoV-2 from infected patient urine; however, it remains unclear whether direct infection of the kidney is responsible for AKI and severity of COVID-19 disease, versus systemic effects resulting from pulmonary distress (12–17).

Human cellular and organoid model systems have played a valuable role in our understanding of SARS-CoV-2 infection mechanisms, interactions with key target organs, and the efficacy of COVID-19 therapeutics, as they provide more complex physiological compositions and behaviors than standard 2D culture (18–24). Human models are particularly valuable because mice are not generally susceptible to SARS-CoV-2 without adaptation to mouse ACE2 (25). Kidney organoids are segmented structures that resemble primitive nephrons, which can be differentiated in vitro from human pluripotent stem cells, including induced pluripotent stem cells and embryonic stem cells (26–29). These organoid cultures contain diverse cell types in patterned, well-differentiated structural arrangements, an advantage over traditional primary cultures that consist primarily of dedifferentiated proximal tubular cells. Because human pluripotent stem cells are immortal, they are readily amenable to genome editing to produce isogenic pairs of mutant and control cell lines, enabling reconstitution of hereditary disease phenotypes such as polycystic kidney disease (PKD) cystogenesis in derived organoids (28). Such cell lines cannot be readily established from primary cultures, which typically senesce rapidly and have a much more limited proliferative potential (24). SARS-CoV-2 can infect kidney organoid cultures, a property that has been leveraged to test candidate therapeutics (19, 22). Because organoids contain multiple cell types, additional experiments are needed to determine which of these cell types are specifically infected by using novel fluorescence-reporter SARS-CoV-2 variants (30–33). The ability to colocalize SARS-CoV-2 infection with markers of apoptosis would provide critical insight into whether infection can produce direct cytotoxic effects simulating AKI, which were not detected in a recent study of somatic-derived kidney spheroids (34). It is also important to further apply this system to compare with clinical cohorts and viral variants of concern and to screen candidate therapeutics for safety and efficacy related to kidney disease.

Application of genome-edited organoids to SARS-CoV-2 presents valuable opportunities to determine mechanisms of viral infection and assess the impact of preexisting disease states (18, 28, 35–37). In this study, we cross-validate SARS-CoV-2’s impact on kidney organoids and clinical data to investigate tropism, mechanism, and therapeutics. These findings provide clear evidence of SARS-CoV-2’s kidney tropism, acute cytopathic effects, systemic and kidney-specific proteomic responses, and efficacy of COVID-19 therapeutics.

## Results

### SARS-CoV-2 infects organoid proximal tubules with pathogenic effects.

**Figure 1:**
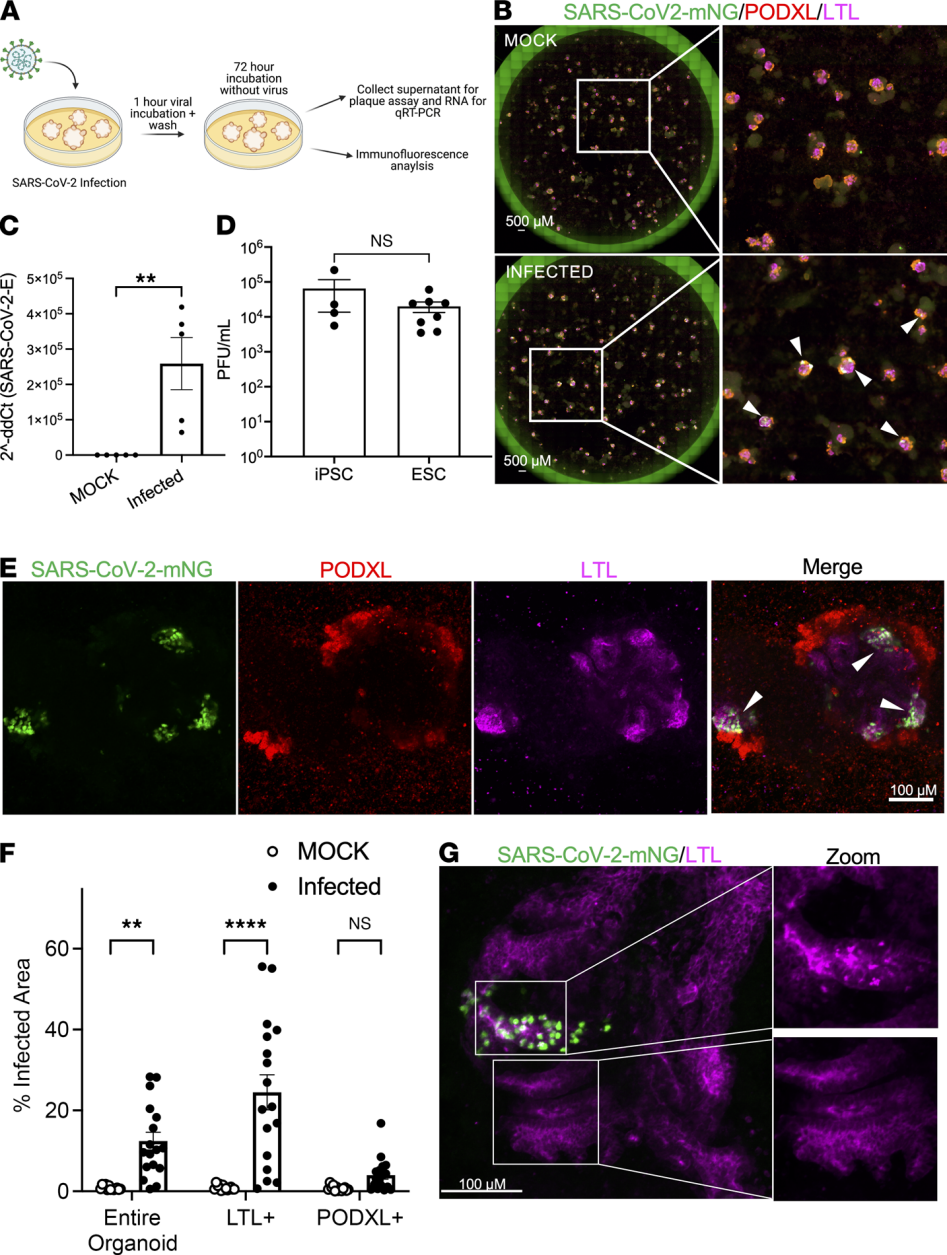
SARS-CoV-2 efficiently infects human kidney organoids with tropism for proximal tubules. (**A**) Schematic of kidney organoid infection protocol. (**B**) Whole-well wide-field immunofluorescence images of iPS cell–derived organoids infected with SARS-CoV-2-mNG. Arrowheads point to infected cells. (**C**) qRT-PCR of SARS-CoV-2 envelope RNA in organoids infected with SARS-CoV-2/WA1 or mock-infected (MOCK). Dots represent a well of organoids. Mean ± SEM, *n* ≥ 1 well of organoids per infection from 4 independent experiments. Mann-Whitney test, **P < 0.01. (**D**) Plaque assays of SARS-CoV-2–infected human kidney organoids derived from iPS cells or ES cells. Dots represent a well of organoids. Mean ± SEM, *n* ≥ 1 well of organoids per infection from 3 independent experiments, respectively. Mann-Whitney test, NS *P* > 0.05. (**E**) Representative confocal immunofluorescence images of organoids infected with SARS-CoV-2-mNG. (**F**) Quantification of infected organoid cellular tropism. Dots represent a single organoid. Mean ± SEM, *n* ≥ 4 organoids per infection from 3 independent experiments. Two-way ANOVA, multiple comparisons, MOCK vs. infected for each respective region; **P < 0.01, ****P < 0.0001, NS *P* > 0.05. (**G**) Representative confocal immunofluorescence images of organoid infected with SARS-CoV-2-GFP, with zoomed images of white boxed areas showing infected (top) versus uninfected (bottom) proximal tubules. Arrowheads indicate areas of disrupted LTL pattern.

To assess the susceptibility of kidney organoid cell types to SARS-CoV-2 infection, we used an adherent differentiation protocol that produces nephron-like epithelial structures surrounded by a monolayer of stromal and endothelial cell types (28, 32). We exposed these human kidney organoid cultures to an MOI of 10 of SARS-CoV-2/WA1 (SARS-CoV-2) and measured infection 72 hours later (Figure 1A). Using SARS-CoV-2 genetically engineered to express mNeonGreen (SARS-CoV-2-mNG), we observed that the fluorescent signal localized in epithelial kidney organoid structures, whereas the surrounding monolayer of stromal and endothelial cells was not infected (Figure 1B) (30). Viral RNA of SARS-CoV-2 was readily detected in infected cultures, indicating that virus had entered cells (Figure 1C). Seventy-two hours after infection, supernatants from organoids exposed to SARS-CoV-2 or SARS-CoV-2-mNG efficiently infected Vero cells and produced viral plaques, demonstrating functional virion production in kidney organoids (Figure 1D and Supplemental Figure 1A; supplemental material available online with this article; https://doi.org/10.1172/jci.insight.154882DS1). Immunofluorescence analysis of nephron markers in organoids exposed to SARS-CoV-2-mNG revealed specific infection of *Lotus tetragonolobus* lectin–positive (LTL^+^) proximal tubules (Figure 1E). The strong binding of LTL to infected cells suggested that these were likely to be proximal rather than distal tubules (33). Podocytes were not generally infected, but individual podocytes (PODXL^+^) infected with SARS-CoV-2-mNG were occasionally observed by confocal microscopy (Supplemental Figure 1B). Similarly, cells resembling the parietal epithelial cells of Bowman’s capsule (CLDN1^+^) were not generally infected (Supplemental Figure 1C) (33, 35). Using semiautomated image analysis quantification, we found that 12.4% of the total organoid area and 24.5% of the total LTL^+^ area were infected, respectively, whereas infection of podocytes (PODXL^+^) was not significant (Figure 1F and Supplemental Figure 2, A–D).

We found that the use of SARS-CoV-2-mNG was vital for establishing the tropism of infection in kidney organoids. In contrast to SARS-CoV-2-mNG, a commercially available GFP-expressing lentivirus pseudotyped for SARS-CoV-2 failed to productively infect kidney organoids or Vero cells (Supplemental Figure 3A). This likely reflects inferior levels of infection by SARS-pseudotyped lentiviruses, compared with native virus (38). In addition, a commercially available antibody raised against SARS-CoV-2 nucleocapsid did not produce specific staining in organoids infected with SARS-CoV-2, but rather showed high background staining levels in stromal cells, necessitating our use of SARS-CoV-2-mNG to evaluate viral tropism (Supplemental Figure 3B).

Close inspection of infected versus noninfected proximal tubules revealed swollen, rounded cells with a disruption of the smooth LTL patterning at the apical plasma membrane showing increased compartmentalization into bright foci (Figure 1G). Immunofluorescence analysis of cleaved caspase-3 was inconclusive because it was difficult to discern in these densely packed, 3D structures (Supplemental Figure 3C). Lactate dehydrogenase (LDH) release was not detectably increased in infected organoid supernatants, consistent with the observation that SARS-CoV-2 treatment was not overtly toxic to these cultures as a whole (Supplemental Figure 3D). These findings reveal specific proximal tubular tropism of SARS-CoV-2 capable of producing replicating virus and disrupting tubular morphology.

### SARS-CoV-2 infects PKD cystic epithelium, causing cytotoxicity.

**Figure 2:**
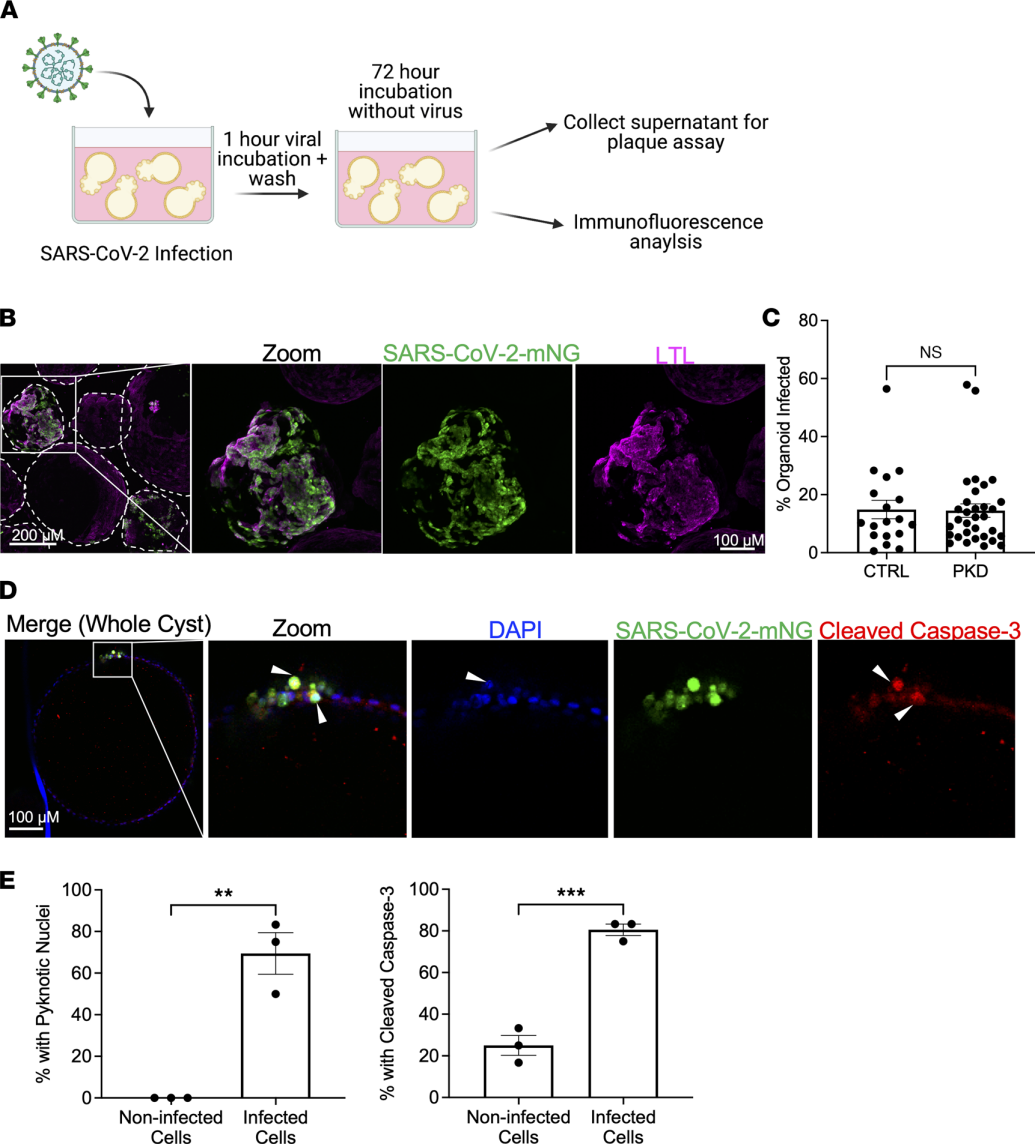
SARS-CoV-2 infects PKD organoid cystic epithelium. (**A**) Schematic of cystic PKD organoid infection protocol. (**B**) Representative confocal immunofluorescence images showing cystic PKD organoids infected with SARS-CoV-2-mNG. Outlines denote independent organoids. (**C**) Quantification of infected organoid area (percent total) of PKD and control (isogenic non-PKD) SARS-CoV-2-mNG–infected cultures. Dots represent a single organoid. Mean ± SEM, *n* ≥ 4 organoids per infection from 3 independent experiments each. Unpaired *t* test, NS *P* > 0.05. (**D**) Representative immunofluorescence images of cystic PKD organoids infected with SARS-CoV-2-mNG, with zoom of cleaved caspase-3 staining and pyknotic nuclei. (**E**) Quantification of pyknotic nuclei and elevated cleaved caspase-3 levels of infected and noninfected cells of infected organoids. Dots represent a biological replicate. Mean ± SEM, *n* ≥ 5 organoids per biological replicate from 3 independent experiments each. Unpaired *t* test, **P < 0.01, ***P < 0.001.

Polycystic kidney disease (PKD) is the most common genetic cause of chronic kidney disease and a possible risk factor for developing severe COVID-19, but these patients are rare, which has impeded large cohort studies (39). In PKD, expansive cysts form from tubular epithelial cells, but whether PKD cysts are susceptible to SARS-CoV-2 infection is unknown. We assessed this with *PKD2–/–* organoids in suspension culture, which form cysts from proximal and distal tubules (Figure 2A) (37). Cystic organoids were infected with SARS-CoV-2 and SARS-CoV-2-mNG and assessed for viral infection and replication via plaque assay and immunofluorescence staining (Figure 2B and Supplemental Figure 4A). A subpopulation of cyst-lining epithelial cells with LTL binding affinity (suggesting a proximal tubular origin) was infected selectively by SARS-CoV-2-mNG and caused cell swelling (Figure 2B and Supplemental Figure 4B). The percentage of infected area per cystic organoid was comparable to that of noncystic organoids (Figure 2C).

Infection-induced apoptosis of cystic PKD epithelium was observed in infected organoids as indicated by significantly increased expression of cleaved caspase-3 and pyknotic nuclei in infected cells, compared with noninfected cells in the same organoid (Figure 2, D and E). These indicators of cytotoxicity were more readily discerned in the thinner layer of cyst-lining epithelial cells than in denser organoid tubular structures without a PKD phenotype (Supplemental Figure 3C). Together, these data indicate that PKD cysts derived from proximal tubules are susceptible to SARS-CoV-2 infection in organoids, and that infection induces apoptosis in cystic epithelium.

### COVID-19^+^ patient urine expresses signatures found in organoids.

**Figure 3:**
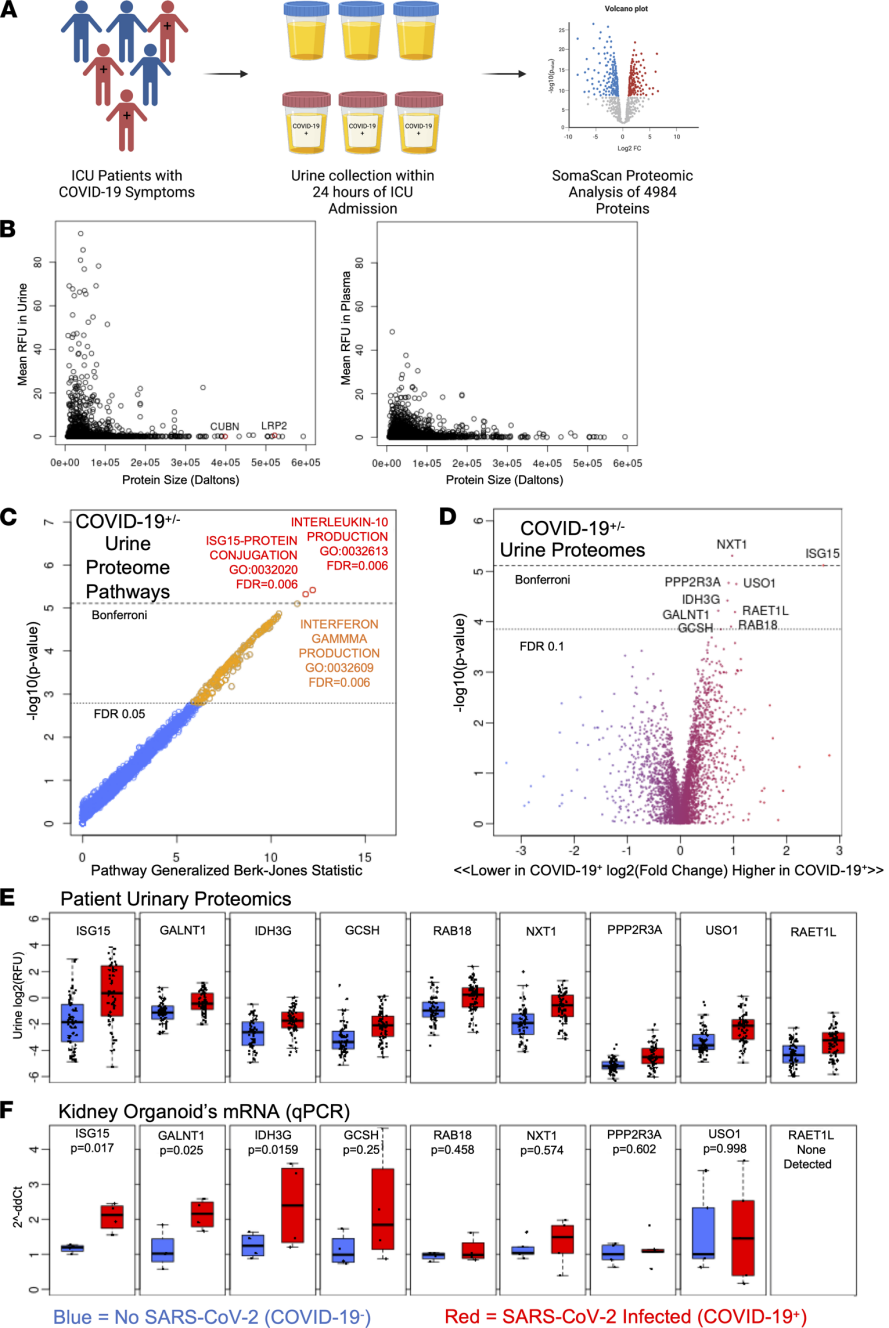
COVID-19^+^ patient urine expresses signatures found in organoids. (**A**) Schematic of patient cohort sample selection and analysis. (**B**) Scatterplots of protein size versus relative fluorescence units (RFU) detected in patient urine and patient blood. (**C**) Gene Ontology pathway analysis of urine proteome reads. Red circles represent pathways that hit Bonferroni significance, orange circles represent pathways that hit an FDR less than 0.05, and blue circles represent nonsignificant pathways. (**D**) Volcano plot of increased and decreased proteins in COVID-19^+^ patient urine compared with COVID-19^–^ patient urine. Dotted lines represent FDR 0.1 and Bonferroni significance cutoffs. (**E**) Upregulated proteomic hits between COVID-19^+^ and COVID-19^–^ patients. (**F**) qRT-PCR of upregulated proteomic hits in organoids infected with SARS-CoV-2/WA1 or mock-infected. Dots represent a well of organoids. Mean ± SEM, *n* ≥ 1 well of organoids per infection from 4 independent experiments. Unpaired *t* test.

To assess physiological relevance of the organoid model, we analyzed urinary proteins that characterize COVID-19 infection in prospectively enrolled critically ill patients with signs and symptoms suggestive of SARS-CoV-2 infection, who were placed under respiratory isolation by the treating physician (Figure 3A) (40). Positivity for COVID-19 was subsequently confirmed by reverse transcriptase PCR (COVID-19^+^), while patients who tested negative provided a symptom-matched, critically ill control group (COVID-19^–^). In patients with an indwelling catheter, we collected urine within 24 hours of intensive care unit (ICU) admission from 61 COVID-19^+^ and 59 COVID-19^–^ patients. COVID-19^+^ and COVID-19^–^ patients were of similar mean age (56 ± 17 years and 54 ± 16 years, respectively), while COVID-19^+^ patients were more likely to be male (75% vs. 54%) and of Hispanic ethnicity (38% vs. 7%) (Table 1). Even though COVID-19^–^ patients had higher rates of chronic kidney disease, coronary artery disease, and congestive heart failure, rates of AKI at the time of study enrollment were similar (58% in COVID-19^+^ and 59% in COVID-19^–^). However, by the time of hospital discharge, receipt of acute renal replacement therapy was 2-fold higher in COVID-19^+^ compared with COVID-19^–^ patients (15% vs. 7%).

Urinary proteomes indicated a negative relationship between protein size and relative abundance (Spearman’s rank correlation, ρ = –0.05), whereas patient plasma exhibited no significant relationship of this kind, likely reflecting the size selectivity of the glomerular filtration barrier (Figure 3B and Supplemental Figure 5A). Proteins too large to be efficiently filtered were nevertheless detected in these urines, including cubilin (CUBN) and megalin (LRP2), receptors that are strongly expressed in the brush border of kidney proximal tubular cells. Thus, urinary proteomes contained both filtered proteins originating from the plasma and nonfiltered proteins originating from the kidneys and urinary tract.

We compared 4984 urinary proteins between these patients with and without COVID-19. Using this urine proteomic data, we performed a Gene Ontology pathway analysis, which identified 207 pathways as significantly differentially abundant with a false discovery rate (FDR) less than 0.05. Two pathways, IL-10 production (*P* = 3.83 × 10^–6^, FDR = 0.006) and interferon-stimulated gene 15 (ISG15) protein conjugation (*P* = 4.79 × 10^–6^, FDR = 0.006), met the Bonferroni correction threshold of *P* < 7.80 × 10^–6^. The third most significant pathway was IFN-γ production (*P* = 8.00 × 10^–6^, FDR = 0.006) (Figure 3C).

Nine individual proteins were significantly higher in COVID-19^+^ patients adjusting for age, sex, and BMI with an FDR less than 0.1 (Figure 3D): ISG15, polypeptide *N*-acetylgalactosaminyltransferase 1 (GALNT1), isocitrate dehydrogenase (NAD^+^) 3 non-catalytic subunit γ (IDH3G), glycine cleavage system protein H (GCSH), Ras-related protein 18 (RAB18), nuclear transport factor 2–like export factor 1 (NXT1), protein phosphatase 2 regulatory subunit B’’ alpha (PPP2R3A), USO1 vesicle transport factor (USO1), and retinoic acid early transcript 1L (RAET1L) (Figure 3E). No individual proteins were found to be downregulated in COVID patient urine at this FDR cutoff. A refined linear regression analysis of the proteomic hits (FDR < 0.05) further identified 23 upregulated proteins, of which the top 9 are the same as described above, and revealed 2 downregulated proteins in COVID-19^+^ patient urine, hypoxanthine phosphoribosyltransferase 1 (HPRT1) and CD5 molecule–like (CD5L) (Supplemental Figure 6A).

Transcripts of 8 of these 9 proteins were increased in kidney organoids infected with SARS-CoV-2 compared with their mock-infected controls, including ISG15 and GALNT1, which were significantly upregulated (Figure 3F). We assessed the cellular origins of the 9 upregulated proteomic hits in a COVID-19^+^ urine single-cell RNA sequencing (scRNA-Seq) database, which revealed expression levels of all the hits in urothelial and proximal tubular cells, and a variety expressed in immune and myeloid cells (Supplemental Figure 6, B and C) (41). Collectively, these expression signatures suggested that SARS-CoV-2 infection can induce an interferon response in kidney epithelial cell types similar in both patients and organoids (42–45).

### SARS-CoV-2 variants show similar rates of infection in kidney organoids.

SARS-CoV-2 is a positive-sense, single-stranded RNA virus that utilizes an RNA-dependent RNA polymerase carrying a high mutation rate, up to 1 million times higher than its hosts’ DNA polymerase (46). Higher mutation rates correlate with enhanced virulence of emerging viral strains and are suggested to produce SARS-CoV-2 viral variants with enhanced infectivity, such as the delta variant (47–49). Formal studies directly comparing infection rates between variants of SARS-CoV-2 are required to draw this conclusion. Moreover, it is unknown whether SARS-CoV-2 variants might exhibit differential tropism for the respiratory tract, which is the primary route of infection, versus extrapulmonary organs, such as the kidney.

**Figure 4:**
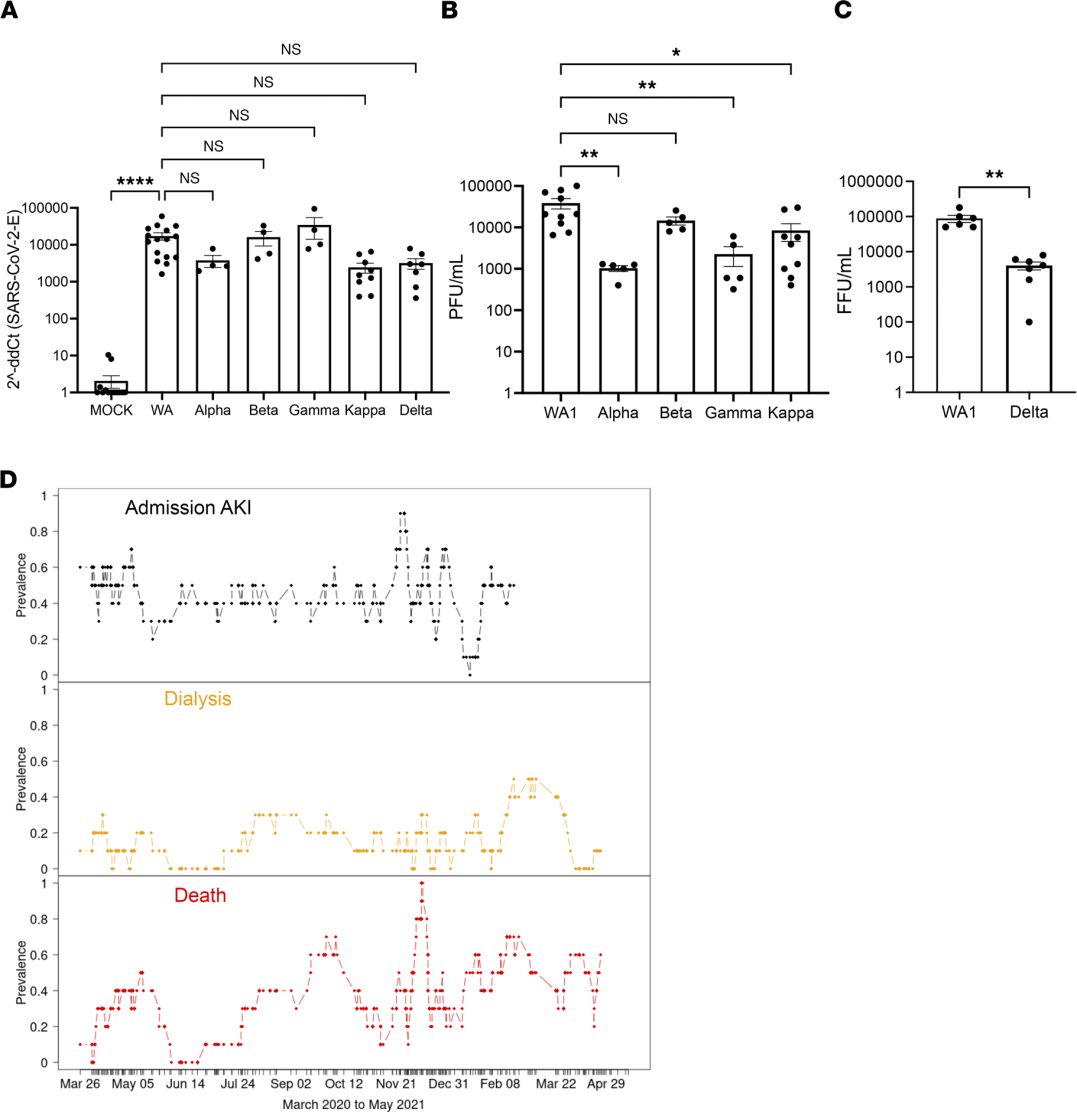
SARS-CoV-2 variants show similar rates of infection in kidney organoids. (**A**) qRT-PCR of SARS-CoV-2 envelope RNA in infected kidney organoid cultures. Dots represent a well of organoids. Mean ± SEM, *n* ≥ 1 well of organoids per infection from 3 independent experiments. One-way ANOVA, Kruskal-Wallis post hoc test, ****P < 0.0001, NS *P* > 0.05. (**B**) Plaque assays of SARS-CoV-2–infected kidney organoids. Dots represent a well of organoids. Mean ± SEM, *n* ≥ 1 well of organoids per infection from 3 independent experiments. One-way ANOVA, Kruskal-Wallis post hoc test, *P < 0.05, **P < 0.01, NS *P* > 0.05. (**C**) Focus-forming assay of SARS-CoV-2–infected kidney organoids. Dots represent a well of organoids. Mean ± SEM, *n* ≥ 1 biological replicates per condition from 3 independent experiments. Mann-Whitney test, **P < 0.01. (**D**) Prevalence of admission AKI, dialysis, and death in COVID^+^ patients over time.

To assess whether SARS-CoV-2 variants exhibit altered viral fitness in kidney organoids, we infected kidney organoids with 6 viral variants: USA-WA1 (WA1), B.1.351-HV001 (beta), B.1.1.7 (alpha), P.1 (gamma), B.1.617.1 (kappa), and B.1.617.2 (delta) (Table 2). Notably, the delta variant we used for infections has a deletion in ORF7a that may influence virulence, although this strain is nevertheless associated with delta outbreaks in human populations (50, 51). Quantitative reverse transcriptase PCR (qRT-PCR) analysis of RNA extracted from infected organoids demonstrated variable levels of detectable SARS-CoV-2 transcript, which were not statistically significant between variants (Figure 4A). Interestingly, supernatants from infected kidney organoids revealed significantly decreased levels of replicating virus from the alpha, gamma, kappa, and delta variants, compared with one of the originally isolated WA1 strains (Figure 4, B and C). Additionally, LDH release from infected kidney organoids was not significantly different between viral strains, nor heightened in comparison with mock-infected controls (Supplemental Figure 7A).

To assess whether rates of admission AKI, dialysis, or death change over time in our patient cohort, we plotted the prevalence, at each patient’s admission, of admission AKI, inpatient dialysis, and in-hospital death over time using that patient and the next nine COVID-19^+^ patients admitted to the ICU (Figure 4D). Death, dialysis, and AKI all had relatively low variance over time, AKI hovering around 40% prevalence, dialysis around 20%, and death around 50% between March 2020 and February 2021. While comprehensive variant sequencing data were unavailable in our COVID-19^+^ patient cohort, in Washington State, the WA1 variant was the predominant viral strain in March 2020, and the alpha, beta, and gamma variants were detected in the United States in January 2021 and kappa and delta detected in March–May of 2021, while rates of AKI and dialysis remained steady in our patient cohort (52–55).

### ACE2 is an essential viral entry pathway for SARS-CoV-2 infection.

**Figure 5:**
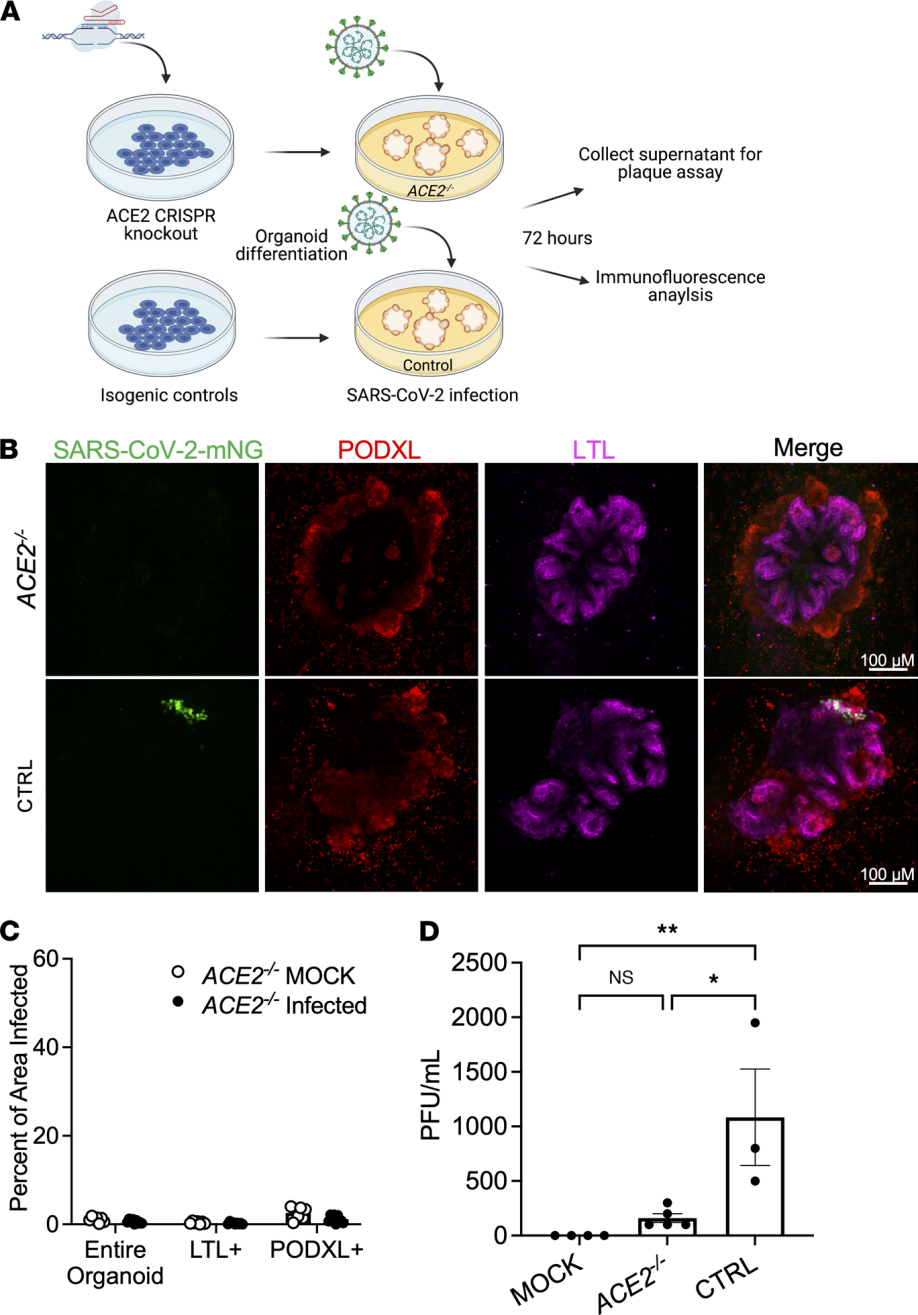
ACE2 is an essential viral entry pathway for SARS-CoV-2 infection of kidney organoids. (**A**) Schematic of ACE2 knockout and infection protocol. (**B**) Representative confocal immunofluorescence images of *ACE2^–/–^* SARS-CoV-2-mNG–infected organoids, compared with isogenic controls. (**C**) Quantification of GFP^+^ area in *ACE2^–/–^* organoids infected with SARS-CoV-2-mNG, compared with mock-treated control. Dots represent a single organoid. *n* ≥ 4 organoids per experiment from 2 experiments. (**D**) Plaque assay of *ACE2^–/–^* and control organoids infected with SARS-CoV-2 or mock-treated. Non-log scale is shown for this figure to emphasize low levels of infection in *ACE2^–/–^* organoids. Dots represent a well of organoids. Mean ± SEM, *n* ≥ 1 well of organoids per infection from 3 independent experiments, using 2 distinct mutant cell lines. One-way ANOVA with Tukey’s post hoc tests, *P < 0.05, **P < 0.01, NS *P* > 0.05.

Susceptibility of kidney organoids to SARS-CoV-2 infection is thought to depend on expression of ACE2, but genetic proof of this is lacking. To assess this, we used genetically modified *ACE2^–/–^* stem cell lines, compared with *ACE2^+/+^* controls (Figure 5A) (18). *ACE2^–/–^* kidney organoids differentiated normally (Supplemental Figure 8A). When exposed to SARS-CoV-2-mNG, however, *ACE2^–/–^* organoids did not express detectable mNG fluorescence, in contrast to *ACE2^+/+^* controls (Figure 5, B and C). Supernatants from *ACE2^–/–^* organoid cultures showed 85% fewer viral particles than supernatants from *ACE2^+/+^* controls and were not significantly different in viral production from mock-infected controls (Figure 5D).

A commercially available antibody raised against ACE2 (antibody 1, aa18–740) exhibited high levels of background in healthy or infected kidney organoids due to the density of these cultures but achieved lower background and higher specificity in cystic PKD organoids, which possess a single-cell epithelium (Supplemental Figure 8, B and C). Antibody 1 appeared more specific in organoids than another commercially available antibody (antibody 2, aa190–230) (Supplemental Figure 8D). The relatively poor performance of these antibodies in kidney organoids was not due to general nonspecificity, as antibody 1 localized specifically to proximal tubular cells in cryosections of human developing kidney tissue, and antibody 2 localized to the proximal tubules of an adult mouse (Supplemental Figure 9, A and B). Therefore, we hypothesized that ACE2 was expressed in organoids but could not be readily detected because of the absence of well-differentiated brush border in these structures, which we have diagnosed previously (28, 33, 56).

Indeed, transcripts encoding both ACE2 and the transmembrane serine protease 2 (TMPRSS2), a coreceptor involved in priming coronavirus for cell entry, were expressed in organoid cultures (41). Interestingly, ACE2 expression trended downward in response to SARS-CoV-2 infection, although not to a statistically significant degree (Supplemental Figure 9C). We previously performed an unbiased scRNA-Seq analysis of these kidney organoid cultures, which indicated a total of 11 distinct cell clusters (32). Among the 6 of these clusters believed to represent kidney cell types, ACE2 was detected specifically in proximal tubules or early proximal tubules, albeit at relatively low expression levels. These same clusters also expressed several other proposed SARS-CoV-2 entry factors, including *TMPRSS2*, *FURIN*, *BSG* (basigin), *ENPEP* (glutamyl aminopeptidase), *ANPEP* (alanyl aminopeptidase), *CTSL* (cathepsin L), and *DPP4* (dipeptidyl-peptidase 4), the latter being particularly abundant in proximal tubules. Many of these other entry factors were expressed in additional cell clusters, such as podocytes or stromal cells, often at greater abundance than in proximal tubules (Supplemental Figure 9D). Overall, these results indicated that SARS-CoV-2 infection of kidney organoids is predominantly mediated by ACE2, and that ACE2 expression may be affected by SARS-CoV-2 infection (57).

### Therapeutics reduce SARS-CoV-2 infection and replication in kidney organoids.

**Figure 6:**
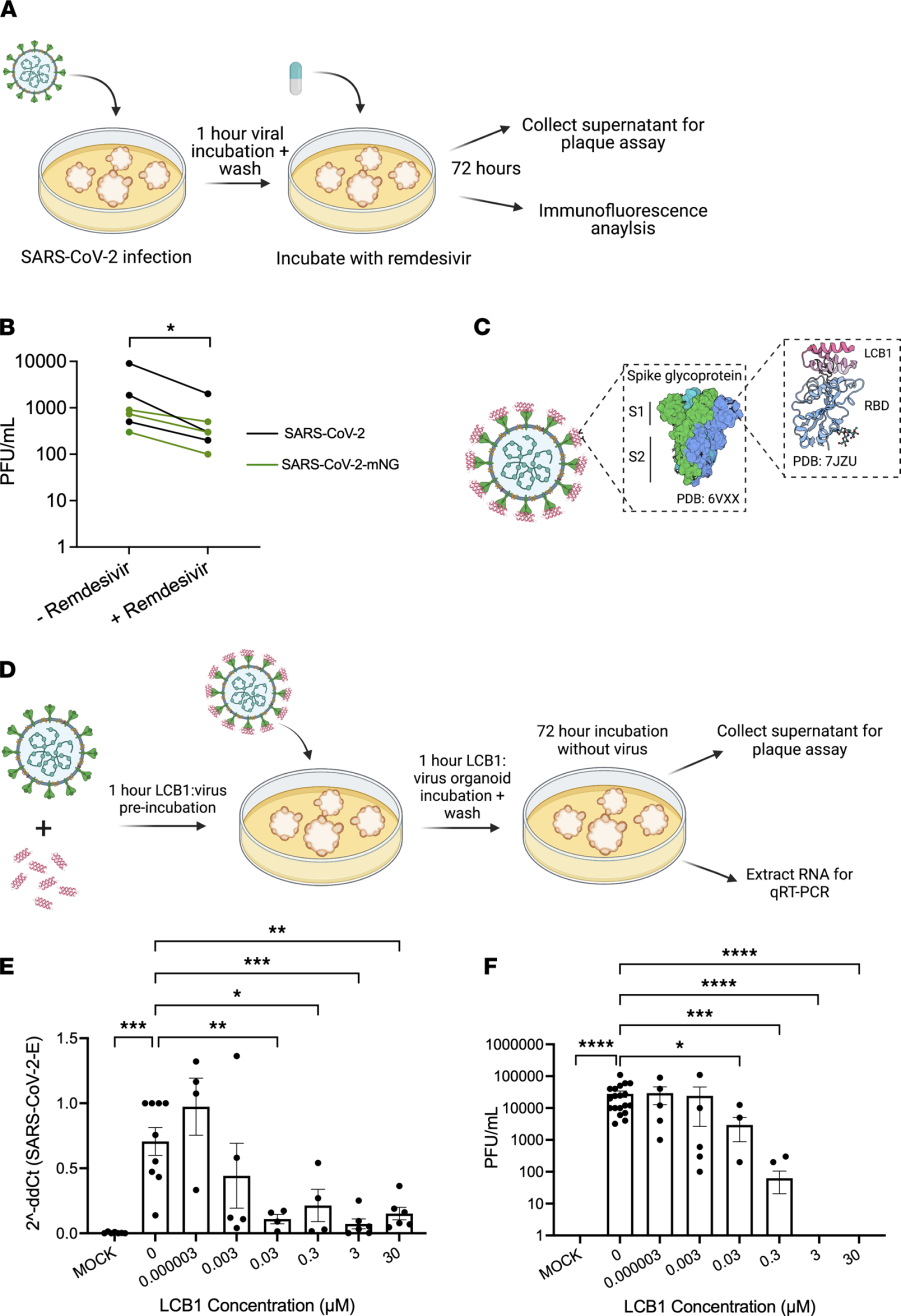
Therapeutic interventions reduce SARS-CoV-2 infection and replication in human kidney organoids. (**A**) Schematic of protocol for SARS-CoV-2 kidney organoid infection with remdesivir treatment. (**B**) Plaque assays of SARS-CoV-2– and SARS-CoV-2-mNG–infected kidney organoids treated with or without remdesivir. Mean ± SEM of 3 independent experiments. Wilcoxon matched-pairs signed rank test, *P < 0.05. (**C**) Schematic of LCB1 binding to spike glycoprotein receptor-binding domain (RBD). (**D**) Schematic of LCB1 viral pretreatment and infection of kidney organoids. (**E**) qRT-PCR expression levels of SARS-CoV-2 envelope RNA in infected kidney organoid cultures, with increasing levels of LCB1 protein preincubated with virus. Dots represent a well of organoids. Mean ± SEM, *n* ≥ 1 well of organoids per infection from 4 independent experiments, 2 iPS and 2 ES, normalized to β-actin. One-way ANOVA, Kruskal-Wallis post hoc test, *P < 0.05, **P < 0.01, ***P < 0.001, NS *P* > 0.05. (**F**) Plaque assays of SARS-CoV-2–infected kidney organoids with increasing levels of LCB1 protein preincubated with virus. Dots represent a well of organoids. Mean ± SEM, *n* ≥ 1 well of organoids per infection from 4 independent experiments, 2 iPS and 2 ES. One-way ANOVA, Kruskal-Wallis post hoc test, *P < 0.05, ***P < 0.001, ****P < 0.0001, NS *P* > 0.05.

The emergence of SARS-CoV-2 has sparked the rapid development of novel therapeutics aimed to block viral infection and replication (58). The nucleotide analog prodrug remdesivir was granted emergency use authorization for the treatment of COVID-19 in May 2020 for its ability to inhibit viral RNA-dependent RNA polymerase (59). While studies have shown that remdesivir treatment in AKI and chronic kidney disease patients is tolerated well, the active metabolite of remdesivir is eliminated by the kidneys and has been reported to increase chances of developing AKI in remdesivir-treated patients (60–62). To investigate the efficacy of remdesivir, we infected kidney organoids with SARS-CoV-2 (WA1) or SARS-CoV-2-mNG, and then treated the infected organoids with a 2 μM dose of remdesivir immediately after infection (Figure 6A). This dose was previously determined to be nontoxic to control organoids in titrations of the compound, whereas doses higher than 8 μM exhibited noticeable toxicity (Supplemental Figure 10A). Comparable levels of toxicity via calcein-AM/propidium iodide staining were noted between control and cystic *PKD2–/–* organoids after 72 hours of treatment (Supplemental Figure 10B). Supernatants from treated and untreated organoids were collected, revealing 71.4% ± 18% reduced replicated virus in the remdesivir-treated organoids (Figure 6B and Supplemental Figure 10C). Immunofluorescence analysis of remdesivir-treated and untreated organoids indicated comparable levels of SARS-CoV-2-mNG infection and intact podocytes and proximal tubules, suggesting that remdesivir treatment did not have an overtly nephrotoxic effect after 72 hours of treatment (Supplemental Figure 10D). These data suggest that remdesivir significantly reduces viral replication of infected kidney organoids and support short-term safety of remdesivir treatment in kidney cells at the efficacious dose, while cautioning that over-dosing of the drug may be counterproductive for the kidneys.

While remdesivir appears to show efficacy in vitro, it is not efficacious in vivo in lowering mortality or reducing infection in COVID-19 patients, necessitating the development of alternatives (63, 64). The de novo–designed protein LCB1 was specifically designed to bind the receptor-binding domain of SARS-CoV-2’s spike protein at picomolar concentrations, has been estimated to have 6-fold greater potency than monoclonal antibodies, and is small enough (6 kDa) to potentially be filtered through the glomerulus, but its efficacy in renal tissues has not yet been tested (Figure 6C) (65, 66). To assess whether LCB1 can block SARS-CoV-2 infection and replication in a kidney-relevant system, we preincubated 0–30 μM of LCB1 with an MOI of 10 of SARS-CoV-2 for 1 hour, and then added the LCB1/virus mixture to kidney organoids (Figure 6D). qRT-PCR analysis of RNA extracted from infected organoids demonstrated an LCB1 dose–dependent decrease of detectable SARS-CoV-2 transcript, with significantly different levels at 0.03 μM and higher (Figure 6E). Supernatants collected from infected organoids were assessed via plaque assay, revealing a dose-dependent decrease in viral particles, starting at ≥0.03 μM, with complete abrogation at the 3 μM and higher doses (Figure 6F). Thus, LCB1 can efficiently block SARS-CoV-2 infection at levels sufficient to prevent viral replication in human kidney organoids.

## Discussion

Renal involvement in SARS-CoV-2 infection and COVID-19 severity is widely acknowledged, but challenges remain in assessing direct and indirect effects of SARS-CoV-2 infection on the kidneys. Using a fluorescent SARS-CoV-2 reporter virus with infectious properties comparable to those of native SARS-CoV-2, we directly demonstrate that the proximal tubules are uniquely infected in these cultures (30). Our experiments using *ACE2^–/–^* kidney organoids rigorously demonstrate the requirement for ACE2-mediated viral entry in kidney organoids, despite their lack of a well-differentiated brush border. A limitation of the current system is that organoids may reflect a more fetal or dedifferentiated state, compared with adult kidneys in vivo, and lack collecting ducts (28, 31, 33, 67). This must be considered when extrapolating findings from organoids to the clinical context.

Similarly, PKD cysts, which feature a more squamous epithelium, contain cells expressing ACE2 and LTL that are susceptible to SARS-CoV-2 infection, which likely derive from proximal tubules (37). The extent of infection observed in cystic organoids is similar to that in control (noncystic) organoids, suggesting that PKD neither enhances nor protects against SARS-CoV-2, consistent with a clinical report in which patients with autosomal dominant PKD did not have significantly increased major COVID-19 outcomes (68). Evidence of cytotoxicity was more readily discerned in PKD cysts than in noncystic tubules, although this may be attributable to improved optics in the cysts. Alternatively, it is possible that increased cell division in the cysts may contribute to a greater susceptibility to injury, as is observed in purified human kidney cells during proliferative expansion in monolayer cultures (34). PKD patients are rare, and none were found among our clinical cohort. Further studies are warranted regarding the potential of SARS-CoV-2 to infect and injure PKD cysts in patients compared with noncystic kidney tissue.

An important question is whether SARS-CoV-2 infection can lead to cytotoxicity in kidney epithelial cells, similar to pulmonary epithelium (69–71). Confocal analysis of infected organoid proximal tubules reveals cell swelling and disrupted LTL marker expression. Cleaved caspase-3 expression and pyknotic nuclei in infected PKD organoids further substantiate infection-induced cytotoxicity. Swelling of the cells might also be suggestive of hypertrophy, which is observed in kidney proximal tubular cells after AKI (72). The higher rates of cell division in our organoid cultures, particularly those with PKD, may contribute to the enhanced sensitivity of these cells to infection-induced injury, compared with spheroids derived from human fetal kidney tissue, which do not show an AKI phenotype (34). Thus, these phenotypes likely reflect an innate, AKI-like program triggered by infection in these human proximal tubular cells.

Our prospective cohort of COVID-19^+^ and COVID-19^–^ patients revealed proteomic upregulation of 9 proteins in COVID-19^+^ patient urine, a signature that was also reflected in kidney organoids and kidney cells in patient urines (41). Upregulation of these genes produces significant differences in interferon and IL-10 Gene Ontology pathways. This supports the hypothesis that AKI in COVID-19 patients may arise as a direct result of kidney infection or a combination of both systemic and kidney-specific response to infection. In vivo, such a scenario would necessitate levels of viremia sufficient to spread infection to kidney cells expressing ACE2. Rates of viremia are quite variable, but recent work suggests that viremia may be higher in critically ill COVID-19 patients (73–75). Once infected, such cells might undergo apoptosis to clear the infection from the kidneys, which would make it difficult to detect after the fact. One limitation of urinary proteomics is that low–molecular weight proteins can be filtered into the urine from the blood; thus detection of a kidney-specific gene expression signature is challenging and requires cross-validation in organoids and other models.

Interestingly, COVID-19 patients with risk allele genotypes for apolipoprotein L1 (APOL1) have been shown to exhibit a collapsing glomerulopathy phenotype (76, 77). Podocytes were rarely infected in our organoids, likely because of the relatively low levels of ACE2 in these cells (33, 78). In previous work, we have shown that organoids at baseline do not express APOL1, but expression can be induced by treatment of the cultures with IFN-γ (79). In this regard, it is interesting that a prominent signature of COVID-19 in our AKI patient cohort relates to IFN-γ signaling, which is a known inducer of APOL1 (79–82). Thus, our study suggests that the collapsing glomerulopathy phenotype in COVID-19^+^ risk allele patients may be a consequence of APOL1 induction in podocytes following IFN-γ exposure, rather than direct infection by the virus. Additional studies are needed to determine the precise impact of APOL1 mutations in SARS-CoV-2–induced collapsing glomerulopathy and the role of IFN-γ in this process.

Remdesivir (Veklury) was granted emergency use authorization by the US Food and Drug Administration in May 2020 for treatment of COVID-19, but more recently, the efficacy of remdesivir for COVID-19 has been challenged (63, 64). The safety and efficacy of remdesivir have not been fully assessed in patients with kidney disease. In animal studies, the kidney was identified as the primary target organ of remdesivir toxicity, and higher levels of remdesivir’s metabolite GS-441524 were found in patients with renal dysfunction, suggesting an added risk with remdesivir treatment in those with chronic kidney disease (83, 84). At a dose of 2 μM, remdesivir was able to significantly reduce viral replication in infected kidney organoids without acutely damaging the epithelial cells, suggesting that a dose providing a balance of safety and efficacy may be achievable. Higher doses, however, showed toxicity in both healthy and control organoids. The C_max_ after injection of a 150 mg dose of remdesivir is 2280 ng/mL, or 3.78 μM concentration in blood, very close to our effective, nontoxic dose used in kidney organoids (83). Our findings suggest that if remdesivir is administered, it should be used with caution to avoid over-dosing, particularly in patients with kidney disease. The novel protein LCB1, designed to prevent SARS-CoV-2’s receptor-binding domain interaction with ACE2, presents a promising new strategy for treating COVID-19 and is small enough to be freely filtered into the urinary space (65). LCB1 doses of 0.03 μM and higher were able to significantly reduce SARS-CoV-2 transcript levels in organoids infected with an MOI of 10, and the number of viral particles in kidney organoid supernatants. These data suggest that LCB1 can efficiently bind to SARS-CoV-2 and prevent viral replication, even in organoids with detectable infections. LCB1 was designed specifically against the receptor-binding domain of the SARS-CoV-2/WA1 variant isolated in 2020. As variants emerge, these spike binder proteins may lose their potency if critical binding domains are mutated, necessitating new designs (85).

Indeed, our data suggest that emerging viral variants may also have distinct capacity for infecting the kidney and may lose that capacity with evolving mutagenesis. While all the variants tested — alpha, beta, gamma, kappa, delta — have reported heightened rates of transmission, controlled experiments are required to measure viral fitness in specific organ systems (86–88). Our data suggest that infection rates of these viral variants are statistically comparable, but replication rates in the alpha, gamma, kappa, and delta strains are significantly decreased, reflecting a potential loss of kidney-specific viral replication fitness in those strains. Notably, the delta strain used in our experiments has a deletion in ORF7a, which may contribute to its lack of virulence due to its inability to antagonize the interferon pathway, although deletions in ORF7a have not been linked with significant functional consequences (30, 89, 90). The SARS-CoV-2-mNG variant we use has the mNG cassette inserted into ORF7a, which demonstrates slightly decreased viral replication, but not significantly so (30). Nevertheless, deletions in delta’s ORF7a have been linked with outbreaks in Australia and Uruguay, suggesting that this strain retains substantial viral fitness (50, 51). The overall organoid infection and replication findings are consistent with clinical observations of decreasing incidence rates of AKI during the pandemic (91). These data could suggest that viral variants may have differential virulence in extrapulmonary organs and, in turn, explain differences in clinical rates of AKI and dialysis during the pandemic. It will be vital to collect both viral sequencing data and clinical data in large cohorts to conclude whether viral variants are linked with changes in rates of AKI.

Collectively, our results reveal that SARS-CoV-2 can directly infect and damage kidney tubular epithelial cells in organoids derived from pluripotent stem cells (92). In this regard, kidney organoid epithelium may model features of lung epithelium in acute respiratory distress syndrome relevant to COVID-19. Data from a controlled human cohort of COVID-19 patients support the physiological relevance of the findings in organoids. The remarkable tropism observed in these organoids, their ability to reveal physiologically relevant injury, and their accessibility to genome editing, together with the availability of urine samples from patients for proteomic analysis, combine to establish a powerful system for studying COVID-19 renal pathophysiology and developing therapeutics (19, 22, 85).

## Methods

### Cell generation

Experiments were performed using wild-type WTC11 induced pluripotent stem (iPS) and H9 embryonic stem (ES) cell lines, and a single *PKD2^−/−^* and 2 *ACE2^–/–^* clones (WTC11 background), generated and characterized as described previously (18, 28). Altogether these represent 2 distinct genetic backgrounds, sexes, and cell types: (a) male WTC11 iPS cells (Coriell Institute Biobank, GM25256) and (b) female H9 ES cells (WiCell, WA09).

### Kidney organoid differentiation

Work with iPS and ES cells was conducted under the approval and auspices of the University of Washington Embryonic Stem Cell Research Oversight Committee. Specific cell lines used in this study are described below and were sourced from commercially available iPS and ES cell lines obtained with informed consent. Stem cell stocks were maintained in mTeSR1 media with daily media changes and passaging using Accutase (STEMCELL Technologies). One thousand to six thousand cells per well were placed in each 24-well plate precoated with 300 μL of DMEM-F12 containing 0.2 mg/mL Matrigel and sandwiched the following day with 0.2 mg/mL Matrigel in mTeSR1 (STEMCELL Technologies) to produce scattered, isolated spheroid colonies. Forty-eight hours after sandwiching, spheroids were treated with 12 μM CHIR99021 (Tocris Bioscience) for 36 hours, then changed to RB (Advanced RPMI + 1× Glutamax + 1× B27 Supplement, all from Thermo Fisher Scientific) and replaced every 3 days thereafter. Organoids were differentiated for 21 days from the time of plating, at which time tubular structures had formed. Gene-edited *PKD2^−/−^* organoids were picked from the adherent plate at day 21, placed in suspension culture with RB media replaced every 3 days until day 30 when cyst growth was prominent (37).

### SARS-CoV-2 generation

All experiments using live virus were performed in the Biosafety Level 3 (BSL-3) facility at the University of Washington in compliance with the BSL-3 laboratory safety protocols (CDC *Biosafety in Microbiological and Biomedical Laboratories*, 5th ed.) and the recent CDC guidelines for handling SARS-CoV-2. Before removal of samples from BSL-3 containment, samples were inactivated by Trizol or 4% paraformaldehyde, and the absence of viable SARS-CoV-2 was confirmed for each sample by plaque assays. SARS-Related Coronavirus 2, Isolate USA-WA1/2020 (SARS-CoV-2), and icSARS-CoV-2mNG (SARS-CoV-2-mNG) were obtained from BEI Resources (NR-52281) and the University of Texas (Galveston, Texas, USA) (30). Isolate 501Y.V2.HV001 (B.1.351) containing E484K/N501Y/D614G mutations along with furin cleavage site point mutation was obtained from Alex Sigal, Africa Health Research Institute (Durban, South Africa), and amplified in Vero-hACE2-TMPRSS2 cells upon reception. Virus stocks generated were titered on VeroE6-TMPRSS2 cells. SARS-CoV-2 isolates hCoV-19/England/204820464/2020 (NR-54000), hCoV-19/Japan/TY7-503/2021 (NR-54982), hCoV-19/USA/CA-SU-15_S02/2021 (NR-55486), and hCoV-19/USA/PHC658/2021 (NR-55611) were obtained through BEI Resources, National Institute of Allergy and Infectious Diseases, NIH, and propagated in Vero cells (USAMRIID). Briefly, Vero cells were maintained in DMEM (Gibco) supplemented with 10% heat-inactivated FBS, 100 U/mL penicillin, and 100 U/mL streptomycin at 37°C in a 5% CO_2_ humidified incubator. To generate virus stock, cells were washed once with DPBS and infected with SARS-CoV-2 in serum-free DMEM. After 1 hour of virus adsorption, the inoculum was replaced with DMEM supplemented with 2% heat-inactivated FBS, and cells were incubated at 37°C in a 5% CO_2_ incubator until about 70% of cells manifested cytopathic effects. The virus was harvested by collection of the culture supernatant followed by centrifugation at 3000*g* for 15 minutes at 4°C to remove the cell debris. Virus titer was then measured by plaque assay on Vero cells (as described below), and stocks were stored at –80°C.

### SARS-CoV-2 titering

Viral preparations and culture supernatant from SARS-CoV-2–infected kidney organoids were titered using a plaque assay. Briefly, 350,000 Vero cells were seeded in 12-well plates and incubated for 1 hour at 37°C with 10-fold dilutions of virus-containing media. A 1:1 solution of 1.8% cellulose suspension in water (MilliporeSigma) and 2× DMEM supplemented with 4% heat-inactivated FBS, l-glutamine, 1× antibiotic-antimycotic (Gibco), and 220 mg/mL sodium pyruvate was layered on top of the cells, followed by incubation at 37°C for 2 days. After fixing with 10% formaldehyde, the cellulose layer was removed and cells were stained with 0.5% crystal violet solution in 20% ethanol. Plaques were counted, and the virus titer in the original sample was assessed as plaque-forming units per milliliter.

### Fusion forming assay

Viral culture supernatants from delta variant supernatants were counted using a fusion-forming assay rather than plaque assay owing to their small size. The protocol was the same as the titering protocol up to the cellulose layer removal step. After cellulose layer removal, fixed cells were washed with FFA wash buffer: 1× PBS with 0.05% Triton X-100 (MilliporeSigma). SARS-CoV-2 Nucleocapsid (Sino Biological, 40143-R019-100; 1:8000) was added in FFA staining buffer: 1× PBS with 1 mg/mL saponin (MilliporeSigma), and incubated overnight at 4°C. The following day, cells were washed 3 times with FFA wash buffer, and then incubated with goat anti-rabbit IgG HRP antibody (Bio-Rad, 1706515; 1:5000) in FFA staining buffer for 2–3 hours at 4°C. Cells were washed 3 times with FFA wash buffer. TrueBlue Substrate (SeraCare) was added to cells, incubated for 10 minutes or until blue spots were visible with minimal background. Cells were then washed with water to quench the reaction, and blue spots were counted and calculated based on the volume of supernatant added as fusion-forming units per milliliter.

### Viral infection with and without therapeutic treatments

SARS-CoV-2 was diluted to the desired MOI in serum-free DMEM and incubated on kidney organoids for 1 hour at 37°C (noninfected MOCK controls were incubated with DMEM only). Organoids were then washed with DPBS and cultured in RB media. Remdesivir (Selleck Chemicals) treatment involved adding 2 μM remdesivir to RB media after 1 hour of viral incubation. LCB1 spike binder was mixed in a 1:1 ratio with diluted virus and incubated for 1 hour at 37°C before being added to organoids.

### Gene expression analysis and viral RNA detection

Infected organoids were washed once with DPBS and incubated with 500 μL per well of Trizol reagent (Invitrogen) for 30 minutes at room temperature. RNA was purified using the Direct-zol RNA Miniprep (Zymo Research). cDNA was synthesized using SuperScript IV Reverse Transcriptase per the manufacturer’s instructions (Thermo Fisher Scientific). Real-time qRT-PCR was performed with PowerUp SYBR Green Master Mix and the following forward and reverse primers: SARS-CoV-2-E: forward GAACCGACGACGACTACTAGC, reverse ATTGCAGCAGTACGCACACA; ACE2: forward CCATCAGGATGTCCCGGAG, reverse TGGAGGCATAAGGATTTTCTCCA; TMPRSS2: forward TCGAAGAACAATATCTGGTGGCT, reverse GATTAGCCGTCTGCCCTCATTT; ISG15: forward GAGAGGCAGCGAACTCATCT, reverse CTTCAGCTCTGACACCGACA; NXT1: forward GTTGTCATCTGTGGATCAGTGAA, reverse CTACAGAGCTAGGGCTGAATGAA; USO1: forward GAAAGAACAGTTGCTCAGGGTTC, reverse TGTTTGTATTTTGCTTCCCCGTC; GALNT1: forward GGCTTGCATTTCTTTTCCTAAAT, reverse TTGCCAACAGACTGCTCTACATA; PPP2R3A: forward CAGGAGGATTTCATCCCTCTACT, reverse TCGAAGTAATTTTTCCACTCCAA; IDH3G: forward CACAAGGCCAACATCATGAAACT, reverse TCCACAATCATGTTCTCGAAGGT; RAB18: forward GAACTTGCAGCAACAATAGGTGT, reverse AACACCCTGTGCACCTCTATAAT; GCSH: forward GGAAAGTGTGAAAGCTGCTAGTG, reverse TCTTGATCAGCCAACCATCTTCA; β-actin: forward GCGAGAAGATGACCCAGATCAT, reverse GGATCTTCATGAGGTAGTCAGTC.

### Immunostaining

Immunostaining followed by confocal microscopy was used to localize various proteins and transporters in the cysts and organoids. Before staining, organoids were fixed in 4% paraformaldehyde for 30 minutes at room temperature. After fixing, samples were washed in PBS, blocked in 5% donkey serum (MilliporeSigma)/0.3% Triton X-100/PBS, incubated overnight in 1% BSA/0.3% Triton X-100/10 μM CaCl_2_/PBS with primary antibodies, washed, incubated with Alexa Fluor secondary antibodies (Invitrogen), washed, and imaged. Primary antibodies or labels included biotinylated LTL (Vector Laboratories, B-1325; 1:500), E-cadherin (Abcam, ab11512; 1:500), hPODXL (R&D Systems, AF1658; 1:500), ACE2 (antibody 1: R&D Systems, AF933, 1:40; antibody 2: Novus, sn0754, 1:100), cleaved caspase-3 (Cell Signaling Technology, 9661S; 1:100), claudin-1 (Abcam, 15098; 1:300),and SARS-CoV-2 Nucleocapsid (Sino Biological, 40143-R019-100; 1:50). Live and dead staining used calcein-AM (Invitrogen) and propidium iodide (Thermo Fisher Scientific) per the manufacturers’ instructions. Fluorescence images were captured using a Nikon A1R inverted confocal microscope with objectives ranging from ×10 to ×60. Note that we were unable to colocalize SARS-CoV-2 with distal tubules (E-cadherin^+^), because of overlap in spectra between the secondary antibody and SARS-CoV-2-mNG.

### Immunohistochemistry

Kidneys from a male 8-week-old C57BL/6J mouse were collected, paraffin-embedded, sectioned, deparaffinized, quenched with 3 volumes of hydrogen peroxide in methanol, subjected to heat-induced epitope retrieval in 1 mM EDTA, and blocked for 1 hour in 1.5% normal goat serum in PBS. Sections were then stained with ACE2 primary antibody (Novus, sn0754; 1:100), washed, and stained with secondary antibody (goat anti–rabbit biotin; Abcam, ab64256) before imaging using an Olympus BX41 microscope at ×40.

### Patient cohort and clinical data collection

The COVID-19 Host Response and Clinical Outcomes (CHROME) study began enrolling critically ill hospitalized patients suspected of SARS-CoV-2 infection in April 2020. This analysis included the first 120 patients enrolled in CHROME with an indwelling bladder catheter for collection of urine. Details of study enrollment have been previously published (40). In brief, subjects were eligible if they were admitted to an ICU for COVID-19 as defined by having symptoms suggestive of SARS-CoV-2 infection (fever, respiratory symptoms including cough/shortness of breath, or sore throat) and 1 of the following: (a) any respiratory support with supplemental oxygen or an oxygen saturation of less than 94% on ambient air; or (b) any chest radiographic abnormality. A case of COVID-19 was defined by a positive RT-PCR for SARS-CoV-2 from a nasopharyngeal swab. The University of Washington IRB approved all human studies (STUDY9763).

### Sample collection, proteomic platform, and quality control

Urine was collected within 24 hours of ICU admission. Peripheral blood was collected into EDTA anticoagulant tubes within 24 hours of ICU admission. Plasma was isolated by centrifugation (10 minutes, 2000*g*, room temperature). All samples underwent 1 freeze-thaw cycle prior to analysis. Proteomic profiling in urine was completed using the SomaScan Platform (Somalogic) that contains 5284 SOMAmer single-stranded DNA aptamers that bind to protein analytes with high specificity. The assays were performed as previously described (93–95). For each sample, the platform reported a relative fluorescence units (RFU) value for each aptamer-protein pair that provides a scale-free measure of protein abundance. The SomaScan Assay is run using 96-well plates, including hybridization normalization control sequences to control for variability in the Agilent readout process. For readout, SOMAmer reagents are hybridized to complementary sequences on a DNA microarray chip and quantified by fluorescence. Fluorescence intensity in the SomaScan assay for each reagent is related to the relative availability of the 3D shape-charge epitope on each protein (the binding site of the SOMAmer reagent) in the original sample. This reflects each protein’s abundance (concentration) and the shape of the protein and the presence of circulating competitors (physiological or a therapeutic antibody). Plate calibration is performed by calculation of the ratio of the calibrator reference RFU value to the plate-specific calibrator replicate median RFU value for each SOMAmer. The resulting ratio distribution is decomposed into a plate scale factor defined by the median of the distribution and a vector of SOMAmer-specific calibration scale factors. Median intra- and interassay coefficients of variation are approximately 5% (96).

### LCB1 protein expression

LCB1 design was made as described previously (69). For expression and purification, briefly, modified pET-29b(+) *E. coli* plasmid encoding LCB1 was used to transform into chemically competent *E. coli* Lemo21 cells (New England Biolabs). Successfully transformed *E. coli* were selected using Studier autoinduction media supplemented with antibiotics. The cells were harvested by spinning at 4000*g* for 10 minutes and then resuspended in a lysis buffer with DNase and protease inhibitors. The cell lysate was sonicated for 4 minutes total (2 minutes 1 time, 10 seconds on/10 seconds off) with an amplitude of 80%. Soluble fraction was clarified by centrifugation at 20,000*g* for 30 minutes and purified by IMAC (Qiagen) followed by size exclusion chromatography (Superdex 75 10/300 GL, GE Healthcare). All protein samples were analyzed with SDS-PAGE with the purity higher than 95%. LCB1 spike binders were validated to neutralize SARS-CoV-2 in Vero cells before use in organoids (data not shown).

### Semiautomated image analysis to quantify SARS-CoV-2-mNG percentage infection

A custom FIJI macro (Supplemental Data 1) was developed to quantify percentage area infected by SARS-CoV-2-mNG within the entire organoid body as well as within PODXL^+^ and LTL^+^ regions. This workflow was designed to provide an unbiased approach to quantifying infection, whereas nuclei-based segmentation of individual cells proved too noisy for accurate quantification because of the 3D nature of the structures. A representative image analysis workflow is presented in Supplemental Figure 2, A–D. For simplicity, quantification was performed on maximum-intensity projections of confocal *Z*-stacks taken through the full thickness of the organoid body. Organoids were manually outlined and the outside signal was cleared to restrict analysis to the organoid body. For each individual replicate, thresholding parameters were empirically chosen to accurately define PODXL^+^ and LTL^+^ regions and applied uniformly to all MOCK and infected organoid images within a paired set. Binary thresholded images were used to define areas exclusively positive for either PODXL or LTL. Histograms of pixel intensity were generated for each organoid within its entire outline and subregions. Histograms were normalized to convert raw pixel counts to percentage of area, such that each organoid contributes equally to statistical analysis. To define a threshold for pixel intensity considered infected, normalized pixel intensity histograms of the entire organoid outline for all MOCK organoids within a particular set were pooled together, and the average and standard deviation (SD) for the pooled data were quantified. Pixels that were greater than 3 SD above average pixel intensity for the pooled MOCK data were defined as infected. Notably, by this criterion for a perfect normal distribution, MOCK organoids would be expected to have 0.15% of pixels defined as infected. The pixel intensity threshold determined from the entire organoid outline was then uniformly applied to both the PODXL- and the LTL-exclusive regions for all organoid images within a set to determine the percentage infection of each region for all organoids.

### SARS-CoV-2-lentiviral transduction

Kidney organoids and Vero cells were incubated with MOI 10 SARS-CoV-2-lentiviral (Amsbio) particles in Opti-MEM reduced serum media (Thermo Fisher Scientific), incubated for 1 hour, washed with 1× PBS, and incubated for 48 hours in RB media before 4% paraformaldehyde fixation.

### Organoid cell type expression analysis

An RDS file of publicly available D18-21 hESC-kidney organoid scRNA-Seq data (Gene Expression Omnibus GSE115986) using previously described clusters was generated using RStudio and Seurat v3 and used to generate a cellxgene (single-cell visualization platform, Chan-Zuckerberg initiative) instance (32). Dot blots representing cell cluster–specific gene expression were generated using the Visualization in Plugin function.

### Data availability and material transfer agreements

All raw data are available upon reasonable request. PKD mutant cell lines used in this study may be obtained from the corresponding author upon request and in accordance with material transfer agreements from the University of Washington and any third party originating sources. Single-cell data sets are searchable via Nephrocell (http://nephrocell.miktmc.org).

### Statistics

#### Organoid data.

Quantification was performed on data obtained from experiments conducted with controls and treatment conditions side by side on at least 3 different occasions (batches) or cell lines (biological replicates). Error bars represent mean ± SEM. Statistical analyses were performed using GraphPad Prism software. Statistical analyses were performed only for experiments with more than 2 replicates. Statistics are plotted in the respective figures, and the tests used are described in the figure legends.

#### Clinical data.

Admission AKI was defined by comparison of the change (Δ) and fold change in concentration from the admission serum creatinine to the lowest serum creatinine measured in the following 28 days of hospitalization. If the drop in serum creatinine was greater than 0.3 mg/dL or greater than 1.5-fold change, the individual was defined as having admission AKI.

#### Protein data normalization.

The RFU value for each aptamer-protein measurement in each sample was scaled by dividing it by the mean of the aptamer-protein RFUs reported in that sample to correct for background variation in RFU. The log_2_ transformation of mean normalized RFU values was used in regression.

#### Protein fold change.

We quantified the differences in protein abundance by fold change calculated as the ratio of normalized RFU values between comparison groups, which was then log_2_-transformed.

#### Protein regression models.

We performed linear regression (Supplemental Data 2) and logistic regression (Supplemental Data 3) analyses to identify differentially abundant log_2_ protein outcomes between COVID^+^ and COVID^–^ patients using the R glm() function. Significant associations were determined after Bonferroni correction (0.05/4984 = 1 × 10^–5^) as well as the false discovery rate (FDR < 0.1 for logistic and < 0.05 for linear) threshold using the Benjamini-Hochberg method. All models were adjusted for age, sex, and BMI. Adjusted log_2_ protein fold changes are the COVID-19^+/–^ beta from the regression model.

#### Pathway analysis.

The Gene Ontology pathway database is available at the Bader laboratory website (http://download.baderlab.org/EM_Genesets/December_11_2020/Human/entrezgene/Human_GOBP_AllPathways_no_GO_iea_December_11_2020_entrezgene.gmt) (97, 98). Gene IDs matching 4141 SomaLogic protein gene names were identified using the EntrezGene ID in the seq_gene.md (hg19, GRCh37.3), and the lists of protein-aptamers corresponding to each pathway were constructed. Pathways with fewer than 3 or more than 500 of the identified protein-aptamer gene names were excluded from analysis, making for 6410 pathways to be analyzed. The generalized Berk-Jones test (GBJ) was used with the mean normalized RFU values of each set of protein-aptamers corresponding to a Gene Ontology pathway. The GBJ has been shown to be an optimal pathway-based test statistic for aggregated sets of genes, DNA variants, and expression values (99–101). A null model adjusting for sex, age, and BMI was used in each pathway test. Gene Ontology term analysis is presented as a list in Supplemental Data 4.

Statistical analyses relating to the analysis of the clinical and proteomic data were conducted in R version 3.6.3.

### Study approval

Animal work was performed in compliance with the strict ethical requirements and regulations of the University of Washington IACUC under a preapproved animal protocol. Human samples were collected as part of the CHROME cohort (University of Washington IRB 9763 and 6878). No individual personal data were included in the study. The IRB provided waiver of consent to participate in this study.

## Author contributions

This was a collaborative body of work that was only possible through unique combinations of expertise from multiple groups. LH generated all kidney organoids. SM and TYH prepared and titered all viruses. LH and SM infected organoids. LH performed and analyzed LDH assays. LH conducted all immunofluorescence staining in organoids and conducted non-automated image analysis. BSF conducted immunofluorescence in human kidney tissue samples. BJ created the semiautomated image analysis script quantifying the infected kidney organoid areas. AK and SM generated *ACE2^–/–^* stem cell lines. SM performed immunohistochemistry on mouse kidney sections. LH and BSF performed remdesivir titrations. LH, SS, and YTZ performed qRT-PCR analyses. SS and YTZ prepared all LCB1 spike mini spike binder proteins. PKB, CM, EDM, and MMW conceived of and designed the clinical study. RM generated the RDS file. FA analyzed cell-gene relationships. IBS performed all proteomic and clinical data analyses. MK, JLH, CEM, HRB, PKB, JH, MG, and BSF supervised the research and secured funding. LH and BSF wrote and edited the first draft of the manuscript. All authors discussed the results and edited the manuscript.

## Supplementary Material

Supplemental data

Supplemental data 1

Supplemental data 2

Supplemental data 3

Supplemental data 4

## Figures and Tables

**Table 1:**
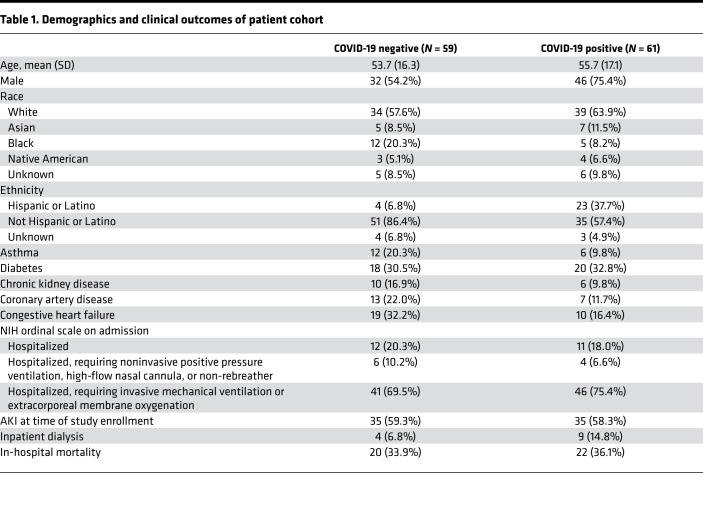
Demographics and clinical outcomes of patient cohort

**Table 2:**
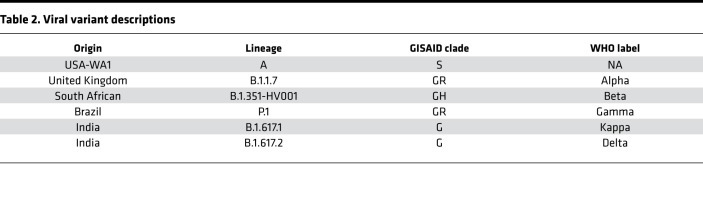
Viral variant descriptions

## Version 1 11/12/2021

In-Press Preview

## Version 2 12/22/2021

Electronic publication
