# Supplementary material for: Cross-validation of SARS-CoV-2 responses in kidney organoids and clinical populations
Source: JCI Insight. 2021 Dec 22;6(24):e154882. doi: 10.1172/jci.insight.154882 (PMC8783682; doi:10.1172/jci.insight.154882)
Supplement: Supplemental data [file jciinsight-6-154882-s184.pdf]

SUPPLEMENTAL FIGURE LEGENDS

S1. A. Plaque assays of SARS-CoV-2 and SARS-CoV-2-mNG infected WTC11 kidney organoids. Dots represent a well of organoids. Mean ± SEM, $n \geq 1$ well of organoids per infection from three independent experiments. Mann-Whitney test, ns $p > 0.05$. B. Immunofluorescent images of a SARS-CoV-2 infected organoid. C. Immunofluorescent images of a SARS-CoV-2 infected organoid.

S2. Representative image analysis workflow for a SARS-CoV-2-mNG infected WTC11 organoid. A. Confocal Z-stacks converted to maximum intensity projections; organoid is then manually outlined with outside signal cleared to restrict analysis to signal within the organoid body. B. Representative multi-channel composite images are split into individual channels for podocalyxin and LTL. User determines threshold parameters for each channel which accurately define podocalyxin- and LTL-positive regions. Uniform thresholding parameters for each channel are applied to all Mock and infected organoid images within a paired set. C. Binary

thresholded images are used to define areas that are exclusively positive for either podocalyxin or LTL. Regions with vertically adjacent distinct cell types artifactually appear co-stained in projection and are excluded from analysis. Podocalyxin- and LTL- exclusive binary images are used as masks to generate images of SARS-CoV-2-mNG pixel intensity within each defined sub-region. D. Histograms of pixel intensity are generated for each organoid within a Mock (not shown) and infected paired set. Histograms normalized based on size of organoid or region to convert raw pixel counts to percent of area, such that each organoid contributes equally to statistical analysis. Normalized histograms of pixel intensity of the organoid outline for all Mock organoids within a particular set are pooled and the average and standard deviation for the pooled data is quantified. Pixels greater than 3 standard deviations above average pixel intensity for the pooled Mock data are defined as infected. By definition for a perfect normal distribution, Mock organoids are expected to have 0.15 % of pixels defined as infected on average. The pixel intensity threshold for infection determined from the organoid outline is uniformly applied to both the podocalyxin- and LTL- exclusive regions for Mock and infected organoid images within a set to determine the percent infection of each area for all organoids.

S3. A. Top panel: Kidney organoids infected with SARS-CoV-2 pseudotyped lentivirus. Bottom panel: Vero cells infected with SARS-CoV-2 pseudotyped lentivirus. B. Immunofluorescence images of SARS-CoV-2 infected organoids. C. Immunofluorescence images of SARS-CoV-2 infected organoids. D. LDH detection assay of infected kidney organoid supernatants 72 hours post SARS-CoV-2 infection. Dots represent a technical replicate. Mean ± SEM, n = 4 technical replicates per condition from two independent experiments. Unpaired t-test, ns $p > 0.05$.

S4. A. Plaque assays of SARS-CoV2 WT and SARS-CoV-2-mNG infected PKD human kidney organoids. Dots represent a well of organoids. Mean ± SEM, n = 1 well of organoids per infection

from three independent experiments. Mann-Whitney test, ns $p > 0.05$. B. Immunofluorescent SARS-CoV2-mNG infected cystic PKD organoids.

S5. A. Scatterplots of protein size vs relative abundance (RFU) of both COVID+ and COVID- patient urine.

S6. A. Volcano plot from linear regression analysis of increased and decreased proteins in COVID-19$^+$ patient urine compared to COVID-19$^-$ patient urine. Dotted lines represent FDR 0.05 and Bonferroni significance cutoffs. B. Reference tSNE plot depicting scRNA-seq data from 13 COVID+ patient urine samples (41). C. Violin plots of urinary cell clusters depicting relative expression of nine proteomic hits from COVID+ patient urine samples (41).

S7. LDH assay of kidney organoid supernatant. Dots represent technical replicate. Mean ± SEM, n = 1-3 technical replicates per condition from three independent experiments per variant. One-way ANOVA, Kruskal-Wallis post-hoc test, ns > 0.05.

S8. A. Immunofluorescent images of ACE2-/- kidney organoid. B. Immunofluorescent images of MOCK and INFECTED WTC11 kidney organoid (Antibody 1). C. Representative immunofluorescence images of ACE2 in cystic PKD organoids infected with SARS-CoV2-mNG (Antibody 1). D. Immunofluorescences image of uninfected WTC11 kidney organoid (Antibody 2).

S9. A. Low- and high-magnification immunofluorescence images of cryosectioned kidney tissue (110 days). Images are representative of kidneys from five different sources (Antibody 1). B. Immunohistochemistry of ACE2 in paraffin section of an adult C57BL6 mouse kidney. Black arrowhead indicates a representative glomerulus (Antibody 2). C. qRT-PCR analysis of ACE2

and TMPRSS2 expression in WTC11 mock and SARS-CoV-2 infected organoids. Dots represent a well of organoids. Mean ± SEM, n ≥ 1 well of organoids per infection from three independent experiments. Unpaired t-test, ns $p > 0.05$. D. Dot blots showing cell type cluster specific expression of SARS-CoV-2 viral entry factors.

S10. A. Representative confocal immunofluorescence and phase images of remdesivir treated kidney organoids. B. Representative confocal immunofluorescence images of live/dead stained *PKD2*$^{-/-}$and *PKD2*$^{+/+}$ remdesivir treated organoids. C. Fold change of SARS-CoV-2 replication of remdesivir treatment organoids compared to DMSO treated controls. Dots represent paired wells of organoids. D. Representative confocal immunofluorescence images of WTC11 SARS-CoV-2 infected organoids with and without 2 μM remdesivir treatment.

S1

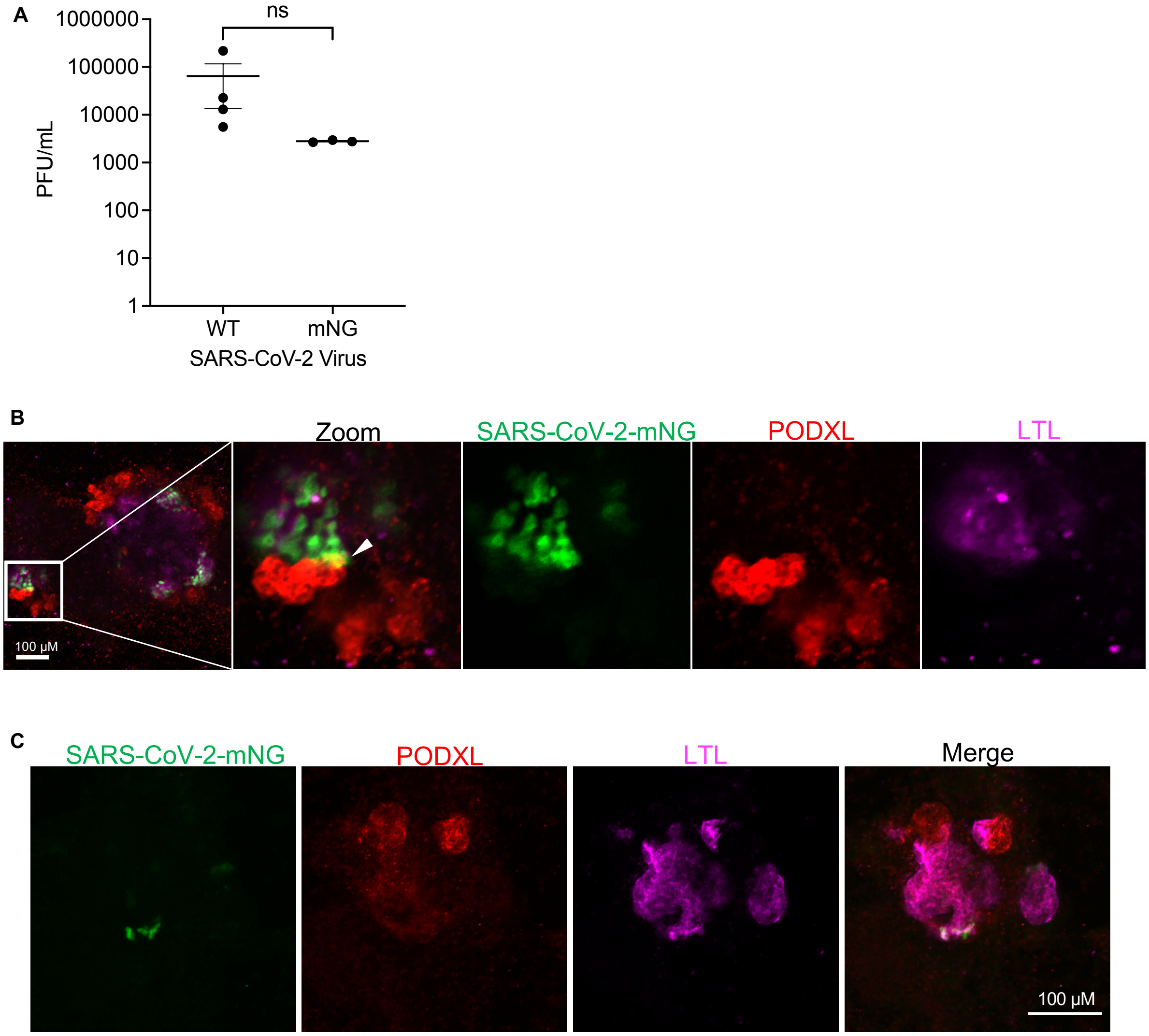
A
ns
1000000
100000
10000
1000
100
10
1
PFU/mL
WT
mNG
SARS-CoV-2 Virus
B
Zoom
SARS-CoV-2-mNG
PODXL
LTL
100 μM
C
SARS-CoV-2-mNG
PODXL
LTL
Merge
100 μM

S2

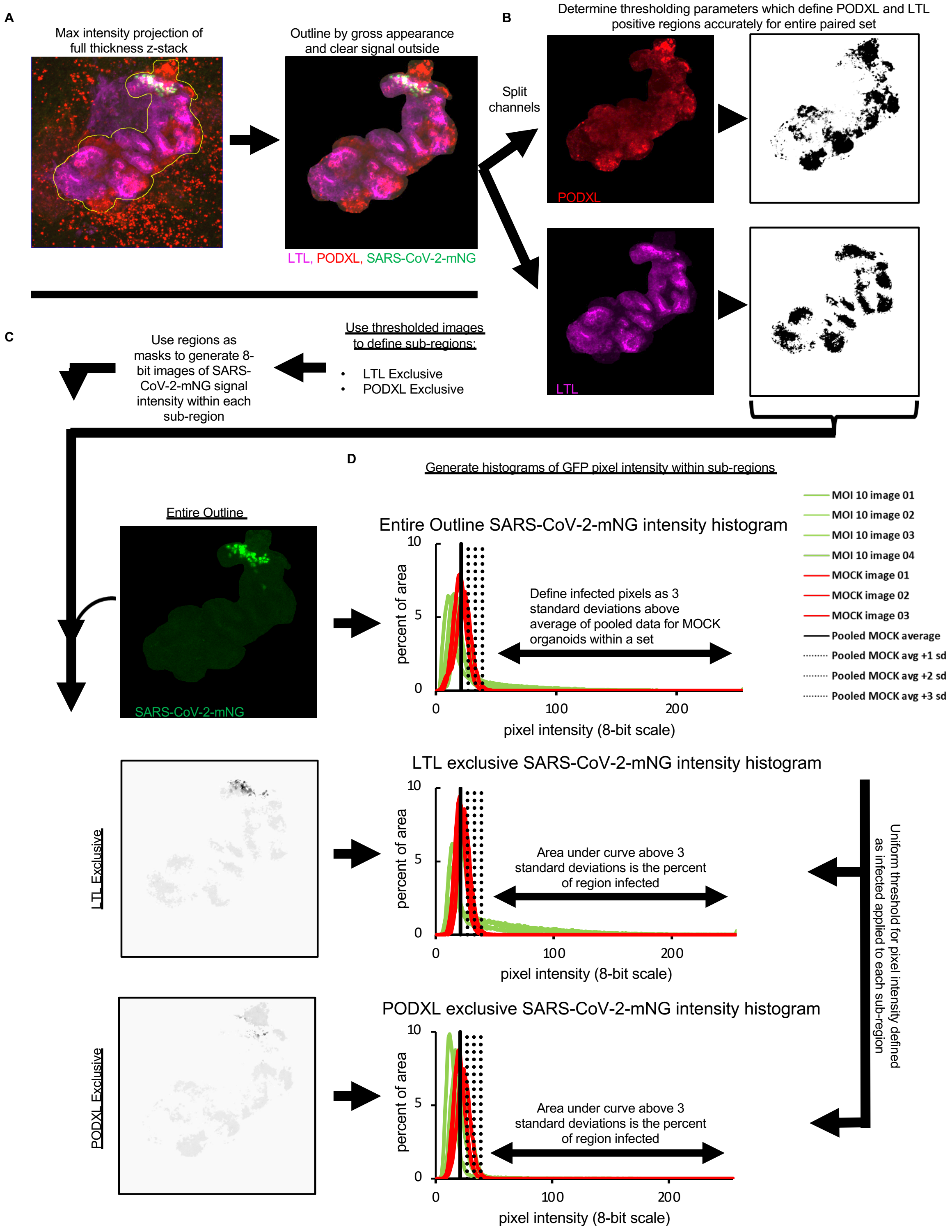
A
Max intensity projection of full thickness z-stack
Outline by gross appearance and clear signal outside
LTL, PODXL, SARS-CoV-2-mNG
B
Determine thresholding parameters which define PODXL and LTL positive regions accurately for entire paired set
Split channels
PODXL
LTL
C
Use thresholded images to define sub-regions:
• LTL Exclusive
• PODXL Exclusive
Use regions as masks to generate 8-bit images of SARS-CoV-2-mNG signal intensity within each sub-region
D
Generate histograms of GFP pixel intensity within sub-regions
Entire Outline
SARS-CoV-2-mNG
Entire Outline SARS-CoV-2-mNG intensity histogram
Define infected pixels as 3 standard deviations above average of pooled data for MOCK organoids within a set
percent of area
pixel intensity (8-bit scale)
MOI 10 image 01
MOI 10 image 02
MOI 10 image 03
MOI 10 image 04
MOCK image 01
MOCK image 02
MOCK image 03
Pooled MOCK average
Pooled MOCK avg +1 sd
Pooled MOCK avg +2 sd
Pooled MOCK avg +3 sd
LTL Exclusive
LTL exclusive SARS-CoV-2-mNG intensity histogram
Area under curve above 3 standard deviations is the percent of region infected
PODXL Exclusive
PODXL exclusive SARS-CoV-2-mNG intensity histogram
Area under curve above 3 standard deviations is the percent of region infected
Uniform threshold for pixel intensity defined as infected applied to each sub-region

S3

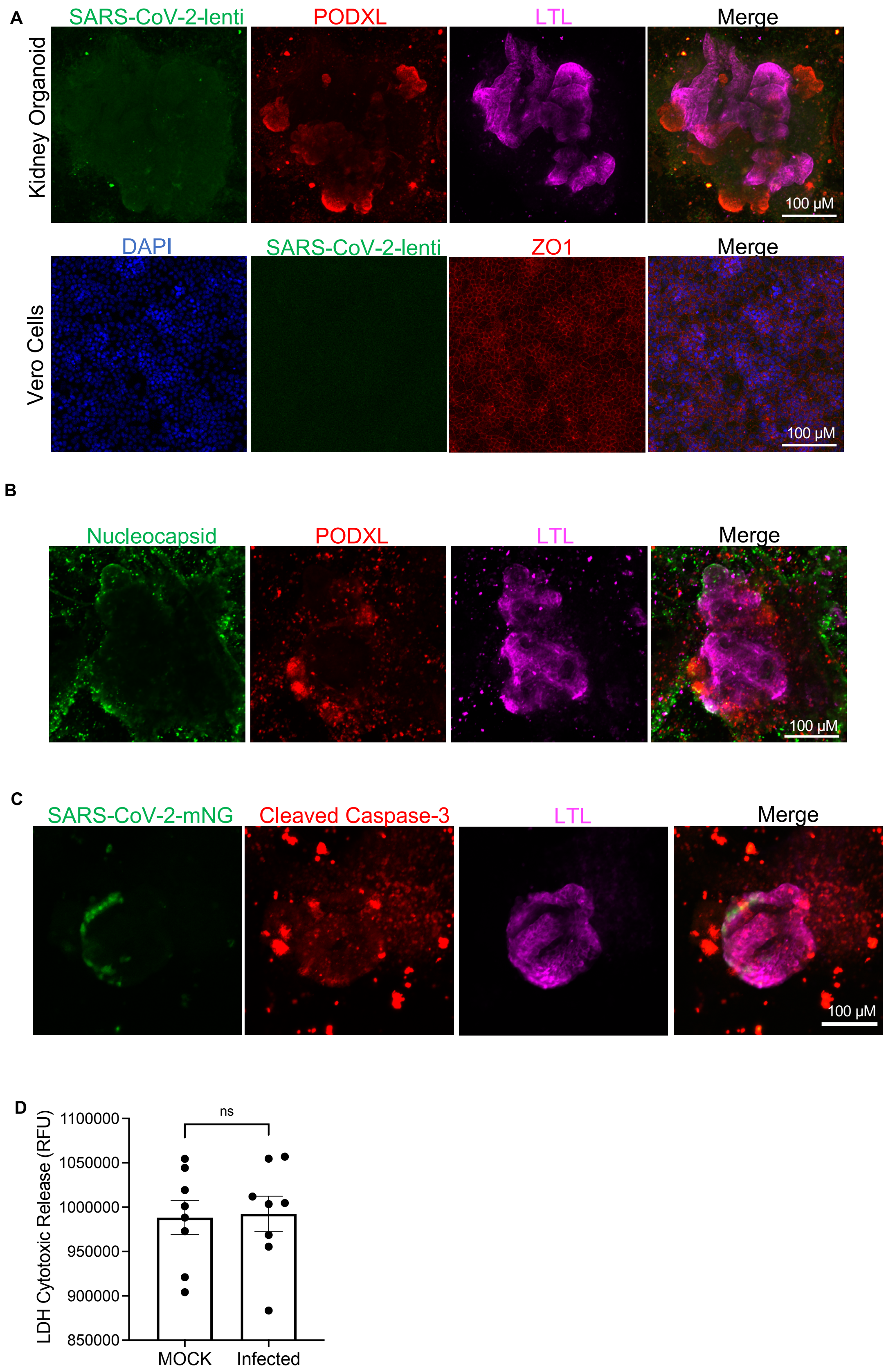

A
SARS-CoV-2-lenti
PODXL
LTL
Merge
Kidney Organoid
100 μM
DAPI
SARS-CoV-2-lenti
ZO1
Merge
Vero Cells
100 μM
B
Nucleocapsid
PODXL
LTL
Merge
100 μM
C
SARS-CoV-2-mNG
Cleaved Caspase-3
LTL
Merge
100 μM
D
LDH Cytotoxic Release (RFU)
1100000
1050000
1000000
950000
900000
850000
ns
MOCK
Infected

S4

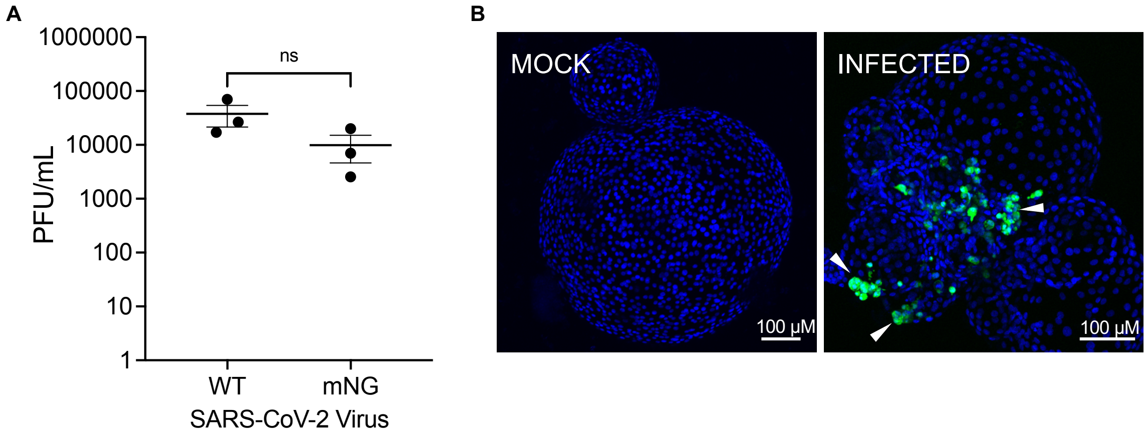

A
1000000
100000
10000
1000
100
10
1
PFU/mL
ns
WT
mNG
SARS-CoV-2 Virus
B
MOCK
INFECTED
100 μM
100 μM

S5

A

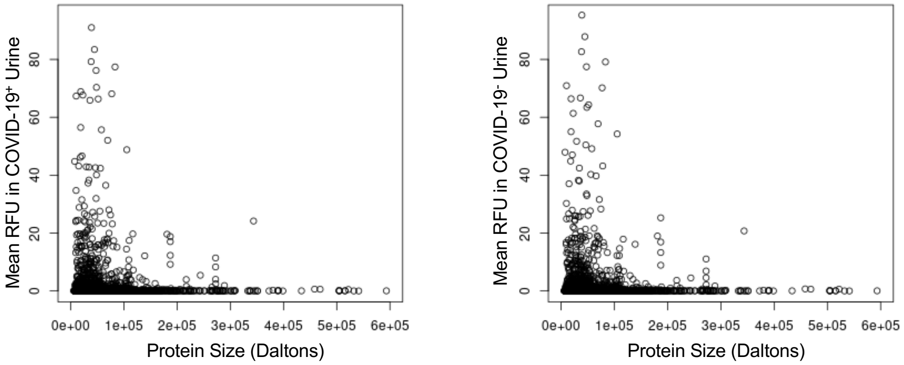

Mean RFU in COVID-19⁺ Urine
0 20 40 60 80
0e+00 1e+05 2e+05 3e+05 4e+05 5e+05 6e+05
Protein Size (Daltons)
Mean RFU in COVID-19⁻ Urine
0 20 40 60 80
0e+00 1e+05 2e+05 3e+05 4e+05 5e+05 6e+05
Protein Size (Daltons)

S6

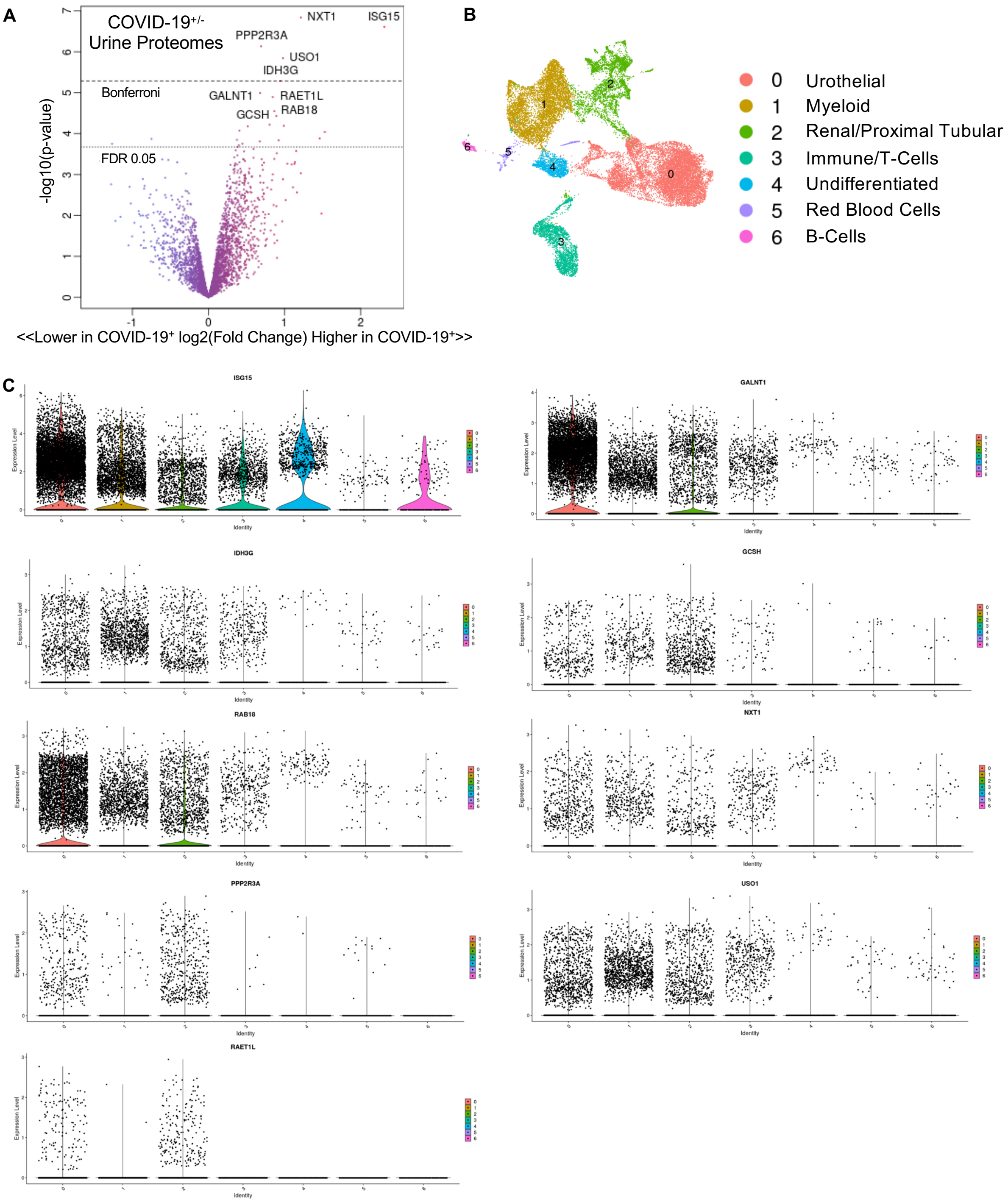

A
COVID-19+/-
Urine Proteomes
Bonferroni
FDR 0.05
NXT1
ISG15
PPP2R3A
USO1
IDH3G
GALNT1
RAET1L
RAB18
GCSH
-log10(p-value)
<<Lower in COVID-19+ log2(Fold Change) Higher in COVID-19+>>
B
0 Urothelial
1 Myeloid
2 Renal/Proximal Tubular
3 Immune/T-Cells
4 Undifferentiated
5 Red Blood Cells
6 B-Cells
C
ISG15
GALNT1
IDH3G
GCSH
RAB18
NXT1
PPP2R3A
USO1
RAET1L
Expression Level
Identity

S7

A

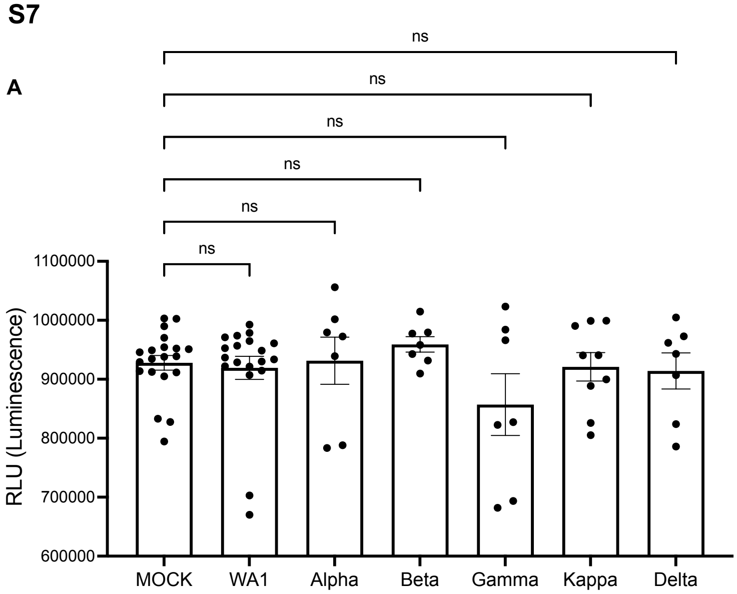

ns
ns
ns
ns
ns
ns
RLU (Luminescence)
1100000
1000000
900000
800000
700000
600000
MOCK
WA1
Alpha
Beta
Gamma
Kappa
Delta

S8

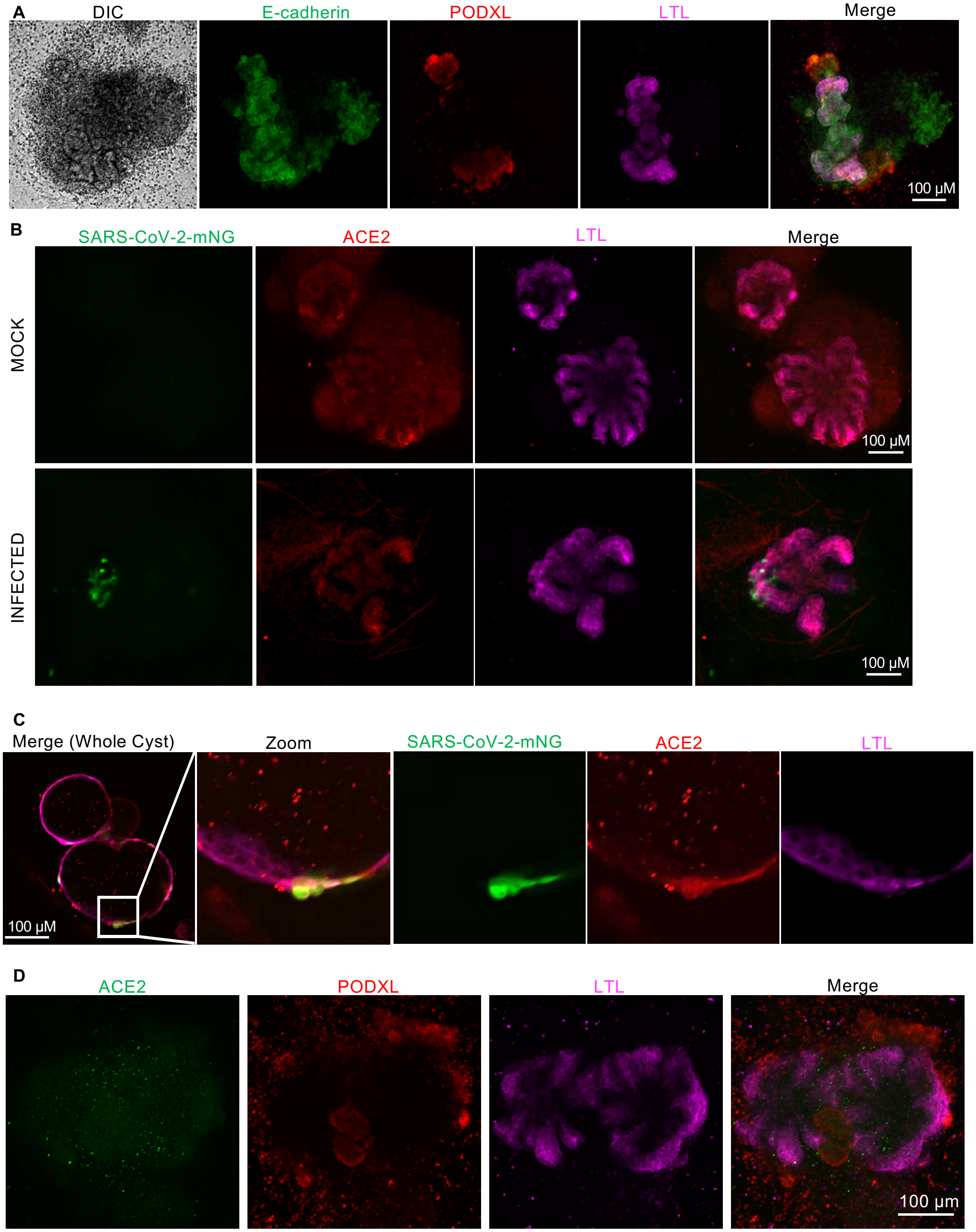
A
DIC
E-cadherin
PODXL
LTL
Merge
100 μM
B
SARS-CoV-2-mNG
ACE2
LTL
Merge
MOCK
100 μM
INFECTED
100 μM
C
Merge (Whole Cyst)
Zoom
SARS-CoV-2-mNG
ACE2
LTL
100 μM
D
ACE2
PODXL
LTL
Merge
100 μm

S9

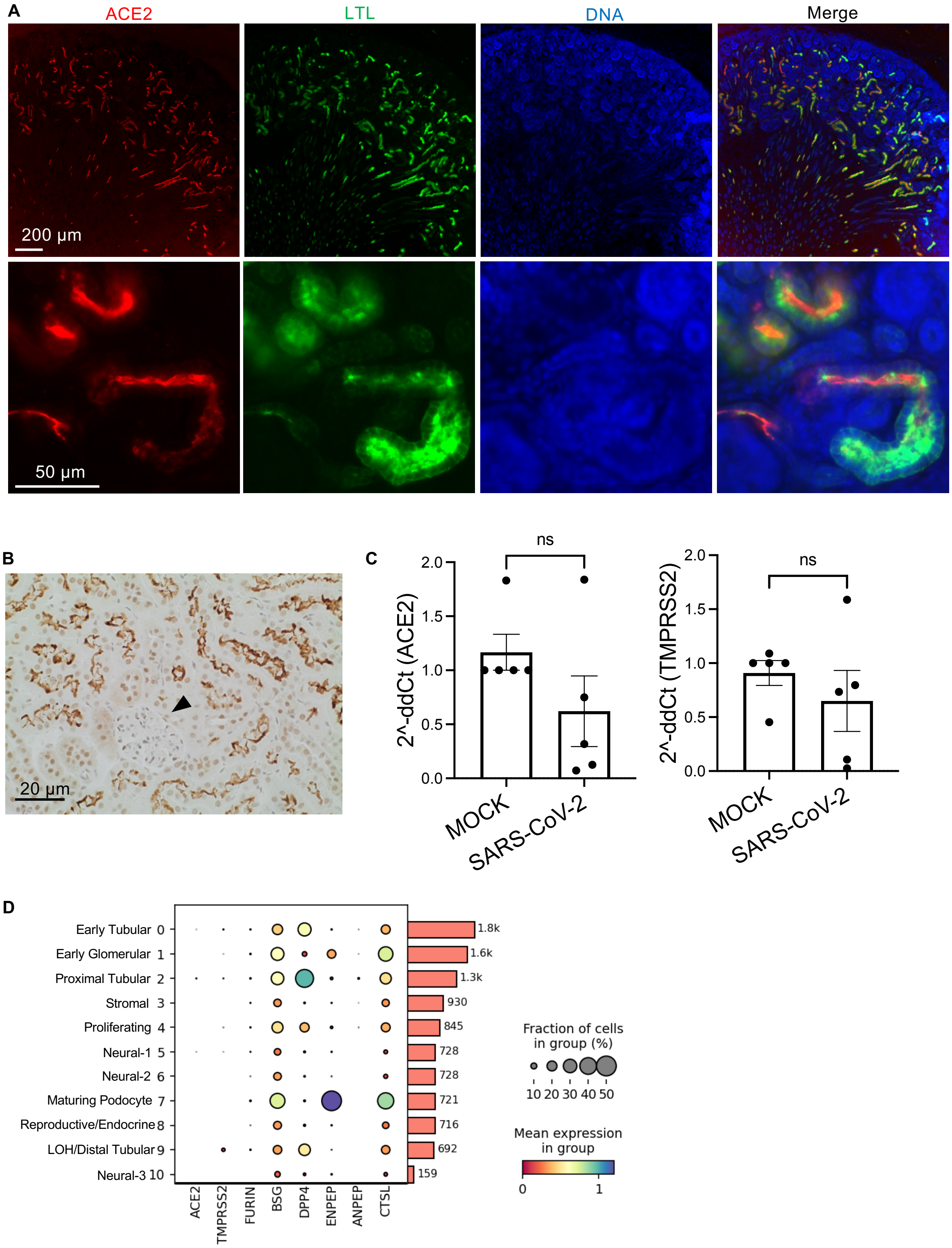

A
ACE2
LTL
DNA
Merge
200 µm
50 µm
B
20 µm
C
ns
2^-ddCt (ACE2)
2^-ddCt (TMPRSS2)
2.0
1.5
1.0
0.5
0.0
MOCK
SARS-CoV-2
D
Early Tubular 0
Early Glomerular 1
Proximal Tubular 2
Stromal 3
Proliferating 4
Neural-1 5
Neural-2 6
Maturing Podocyte 7
Reproductive/Endocrine 8
LOH/Distal Tubular 9
Neural-3 10
ACE2
TMPRSS2
FURIN
BSG
DPP4
ENPEP
ANPEP
CTSL
1.8k
1.6k
1.3k
930
845
728
728
721
716
692
159
Fraction of cells in group (%)
10 20 30 40 50
Mean expression in group
0
1

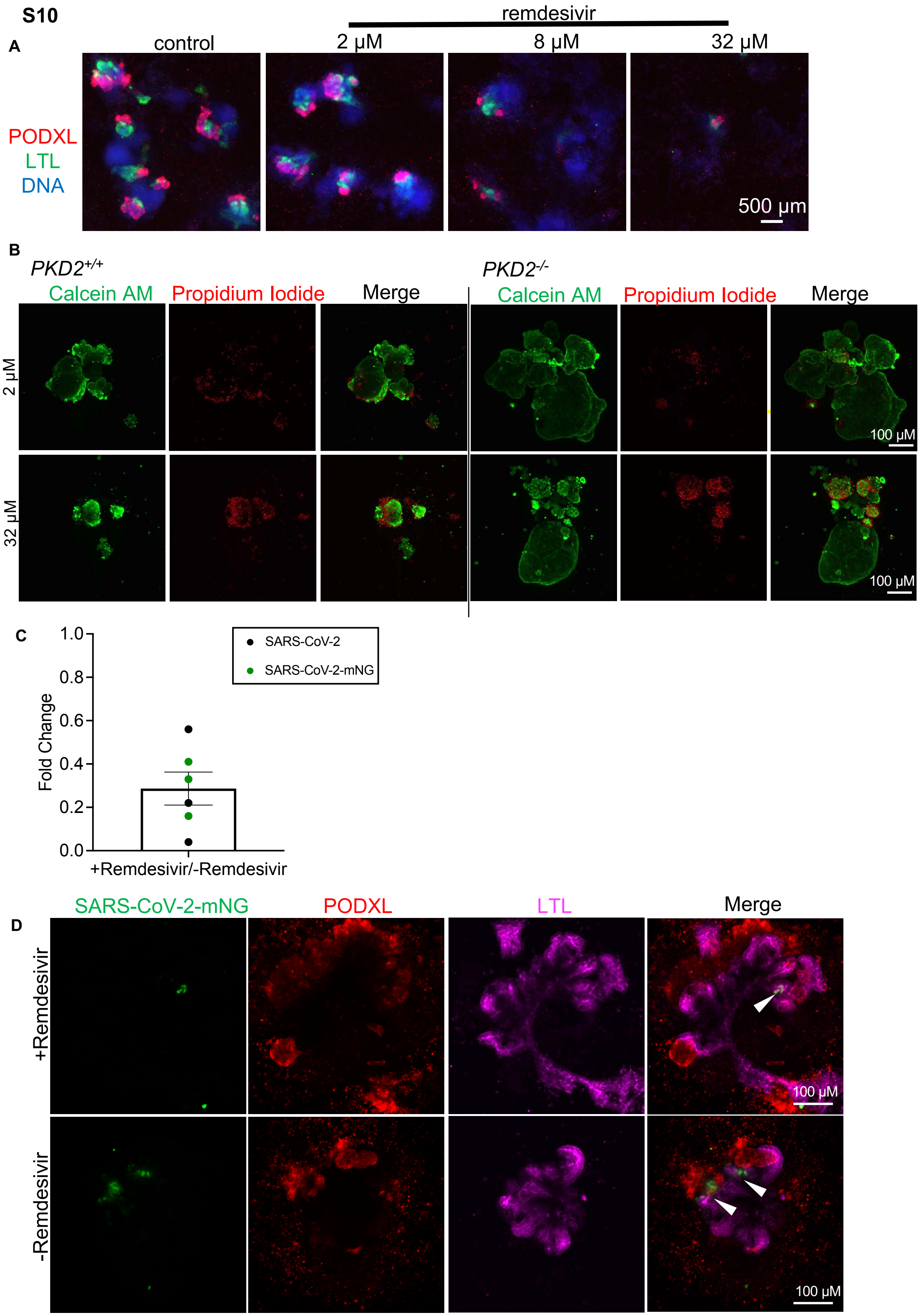
S10
A
remdesivir
control
2 µM
8 µM
32 µM
PODXL
LTL
DNA
500 µm
B
PKD2+/+
PKD2-/-
Calcein AM
Propidium Iodide
Merge
2 µM
32 µM
100 µM
C
Fold Change
1.0
0.8
0.6
0.4
0.2
0.0
+Remdesivir/-Remdesivir
SARS-CoV-2
SARS-CoV-2-mNG
D
SARS-CoV-2-mNG
PODXL
LTL
Merge
+Remdesivir
-Remdesivir
100 µM
