# Supplementary material for: Cross-validation of SARS-CoV-2 responses in kidney organoids and clinical populations
Source: JCI Insight. 2021 Dec 22;6(24):e154882. doi: 10.1172/jci.insight.154882 (PMC8783682; doi:10.1172/jci.insight.154882)
Supplement: Supplemental data 1 [file jciinsight-6-154882-s185.docx]

//To use this FIJI script, copy and paste it into Plugins > New > Macro and set language to IJ1 Macro

//This script works on a directory of multi-channel, single plane (or max-projection) images.

//The user will need to define which particular channel is for which stain.

//Thresholding parameters for podocyte and tubule regions need to be user defined and the script edited for each particular replicate to obtain the most accurate results.

//potentially useful commands and notes have been left in the script and deactivated for ease of adaptation

//selectWindow("Results");

//run("Close");

dir = getDirectory("Choose a Directory");

Newdir1 = dir + "/Podomask/";

Newdir2 = dir + "/LTLmask/";

Newdir3 = dir + "/Organoidoutline/";

Newdir4 = dir + "/overlapmask/";

Newdir5 = dir + "/outsidepodoandLTLmask/";

Newdir6 = dir + "/organoidarea/";

Newdir7 = dir + "/LTLthreshold/";

Newdir8 = dir + "/podothreshold/";

File.makeDirectory(Newdir1);

File.makeDirectory(Newdir2);

File.makeDirectory(Newdir3);

File.makeDirectory(Newdir4);

File.makeDirectory(Newdir5);

File.makeDirectory(Newdir6);

File.makeDirectory(Newdir7);

File.makeDirectory(Newdir8);

list = getFileList(dir);

for(i=0; i<list.length; i++) {

if (endsWith(list[i], ".tif")){

open(dir+list[i]);

imgName = getTitle();

//imgName = "MAX_"+Name;

//baseName=File.nameWithoutExtension();

//run("Clear Outside");

//turn off undesired channels in RBG outlined image

//run("Z Project...", "projection=[Max Intensity]");

//close(Name);

selectWindow(imgName);

run("Clear Outside");

Stack.setChannel("1");

setMinAndMax(0, 4095);

Stack.setChannel("2");

setMinAndMax(-5, 4095);

Stack.setChannel("3");

setMinAndMax(0, 4095);

Stack.setChannel("4");

setMinAndMax(0, 4095);

//Stack.setChannel("5");

//setMinAndMax(0, 4095);

Stack.setActiveChannels("11110");

run("RGB Color");

selectWindow(imgName);

run("Split Channels");

colorimage=imgName+" (RGB)";

//define which channel is for which region

podochannel="C3-"+imgName;

LTLchannel="C4-"+imgName;

covidchannel="C2-"+imgName;

//convert covid channel to 8-bit image

selectWindow(covidchannel);

run("8-bit");

saveAs("tiff", Newdir6 + "organoid area " + imgName);

rename("covidchannel");

//create mask of podo channel

selectWindow(podochannel);

//run("Enhance Contrast...", "saturated=0.5 normalize");

setAutoThreshold("Default dark no-reset");

//run("Threshold...");

//Define subjectively chosen threshold criteria which appear to accurately predict podocyte area for all images within a paired Mock and infected set

setThreshold(200, 65535);

run("Convert to Mask");

saveAs("tiff", Newdir8 + "Podothreshold " + imgName);

//create mask of LTL channel

selectWindow(LTLchannel);

//run("Enhance Contrast...", "saturated=0.5 normalize");

setAutoThreshold("Default dark no-reset");

//run("Threshold...");

//Define subjectively chosen threshold criteria which appear to accurately predict pod area for all images within a paired Mock and infected set

setThreshold(100, 65535);

run("Convert to Mask");

saveAs("tiff", Newdir7 + "LTLthreshold " + imgName);

podochannel="Podothreshold " + imgName;

LTLchannel="LTLthreshold " + imgName;

imageCalculator("AND create", podochannel, LTLchannel);

rename(imgName+"overlaparea");

overlap=imgName+"overlaparea";

imageCalculator("Subtract create", podochannel, overlap);

rename("podoarea");

//podoarea="Result of "+podochannel;

imageCalculator("Subtract create", LTLchannel, overlap);

rename("LTLarea");

//LTLarea="Result of "+LTLchannel;

imageCalculator("AND create", "podoarea", "covidchannel");

saveAs("tiff", Newdir1 + "Podomask " + imgName);

imageCalculator("AND create", "LTLarea", "covidchannel");

saveAs("tiff", Newdir2 + "LTLmask " + imgName);

imageCalculator("AND create", overlap, "covidchannel");

saveAs("tiff", Newdir4 + "overlapmask " + imgName);

imageCalculator("Subtract create", "covidchannel", podochannel);

imageCalculator("Subtract create", "Result of covidchannel", LTLchannel);

saveAs("tiff", Newdir5 + "outsidepodoandLTLmask " + imgName);

selectWindow("podoarea");

run("Outline");

run("Cyan");

run("Flatten");

//run("Invert");

rename("podooutline");

selectWindow("LTLarea");

run("Outline");

//run("Cyan");

run("Flatten");

run("Invert");

rename("LTLoutline");

selectWindow(overlap);

run("Outline");

run("Yellow");

run("Flatten");

//run("Invert");

rename("overlapoutline");

imageCalculator("Transparent-zero create", colorimage, "podooutline");

imageCalculator("Transparent-zero create", "Result of "+colorimage, "LTLoutline");

imageCalculator("Transparent-zero create", "Result of Result of "+colorimage, "overlapoutline");

selectWindow("Result of Result of Result of "+colorimage);

saveAs("JPEG", Newdir3 + "Outlined " + imgName);

while (nImages>0) {

selectImage(nImages);

close("*");

}

}

}

//hisogram generation for Newdir1

run("Input/Output...", "jpeg=85 gif=-1 file=.csv use_file copy_row save_column");

list1 = getFileList(Newdir1);

nBins = 256;

//check for an existing excel file and open it as a results table, ready to append data to it

if (File.exists(Newdir1+"excel file.csv")==true){

open(Newdir1+"excel file.csv");

Table.showRowNumbers(false);

updateResults();

} else{

for (x=0; x<nBins; x++) {

setResult("Value", x, x);

}

}

for (i=0; i<list1.length; i++) {

open(Newdir1+list1[i]);

run("8-bit");

getHistogram(values, counts, nBins);

imageNum = i+1;

Table.setColumn("Count for image "+getTitle(), counts);

updateResults(); //probably faster to do this outside of the loop but the table will not save outside of the loop for some reason

Table.save(Newdir1+"excel file.xls"); //again, I should be saving outside of the loop, but the table is temporary (as if a nested variable!?)

selectWindow(list1[i]);

run("Close");

}

selectWindow("Results");

run("Close");

//hisogram generation for Newdir2

run("Input/Output...", "jpeg=85 gif=-1 file=.csv use_file copy_row save_column");

list2 = getFileList(Newdir2);

nBins = 256;

if (File.exists(Newdir2+"excel file.csv")==true){

open(Newdir2+"excel file.csv");

Table.showRowNumbers(false);

updateResults();

} else{

for (x=0; x<nBins; x++) {

setResult("Value", x, x);

}

}

for (i=0; i<list2.length; i++) {

open(Newdir2+list2[i]);

run("8-bit");

getHistogram(values, counts, nBins);

imageNum = i+1;

Table.setColumn("Count for image "+getTitle(), counts);

updateResults();

Table.save(Newdir2+"excel file.xls");

selectWindow(list2[i]);

run("Close");

}

selectWindow("Results");

run("Close");

//hisogram generation for Newdir4

run("Input/Output...", "jpeg=85 gif=-1 file=.csv use_file copy_row save_column");

list1 = getFileList(Newdir4);

nBins = 256;

//check for an existing excel file and open it as a results table, ready to append data to it

if (File.exists(Newdir1+"excel file.csv")==true){

open(Newdir4+"excel file.csv");

Table.showRowNumbers(false);

updateResults();

} else{

for (x=0; x<nBins; x++) {

setResult("Value", x, x);

}

}

for (i=0; i<list1.length; i++) {

open(Newdir4+list1[i]);

run("8-bit");

getHistogram(values, counts, nBins);

imageNum = i+1;

Table.setColumn("Count for image "+getTitle(), counts);

updateResults(); //probably faster to do this outside of the loop but the table will not save outside of the loop for some reason

Table.save(Newdir4+"excel file.xls"); //again, I should be saving outside of the loop, but the table is temporary (as if a nested variable!?)

selectWindow(list1[i]);

run("Close");

}

selectWindow("Results");

run("Close");

//hisogram generation for Newdir5

run("Input/Output...", "jpeg=85 gif=-1 file=.csv use_file copy_row save_column");

list2 = getFileList(Newdir5);

nBins = 256;

if (File.exists(Newdir5+"excel file.csv")==true){

open(Newdir5+"excel file.csv");

Table.showRowNumbers(false);

updateResults();

} else{

for (x=0; x<nBins; x++) {

setResult("Value", x, x);

}

}

for (i=0; i<list2.length; i++) {

open(Newdir5+list2[i]);

run("8-bit");

getHistogram(values, counts, nBins);

imageNum = i+1;

Table.setColumn("Count for image "+getTitle(), counts);

updateResults();

Table.save(Newdir5+"excel file.xls");

selectWindow(list2[i]);

run("Close");

}

selectWindow("Results");

run("Close");

//hisogram generation for Newdir6

run("Input/Output...", "jpeg=85 gif=-1 file=.csv use_file copy_row save_column");

list1 = getFileList(Newdir6);

nBins = 256;

//check for an existing excel file and open it as a results table, ready to append data to it

if (File.exists(Newdir6+"excel file.csv")==true){

open(Newdir6+"excel file.csv");

Table.showRowNumbers(false);

updateResults();

} else{

for (x=0; x<nBins; x++) {

setResult("Value", x, x);

}

}

for (i=0; i<list1.length; i++) {

open(Newdir6+list1[i]);

run("8-bit");

getHistogram(values, counts, nBins);

imageNum = i+1;

Table.setColumn("Count for image "+getTitle(), counts);

updateResults(); //probably faster to do this outside of the loop but the table will not save outside of the loop for some reason

Table.save(Newdir6+"excel file.xls"); //again, I should be saving outside of the loop, but the table is temporary (as if a nested variable!?)

selectWindow(list1[i]);

run("Close");

}

selectWindow("Results");

run("Close");
